# Supplementary material for: Functional crosstalk between histone H2B ubiquitylation and H2A modifications and variants
Source: Nat Commun. 2018 Apr 11;9:1394. doi: 10.1038/s41467-018-03895-5 (PMC5895630; doi:10.1038/s41467-018-03895-5)
Supplement: Supplementary file 1 — Supplementary Information [file 41467_2018_3895_MOESM1_ESM.pdf]

Supplemental Information

Functional Crosstalk between Histone H2B Ubiquitylation and H2A Modifications and Variants

Wojcik et al.

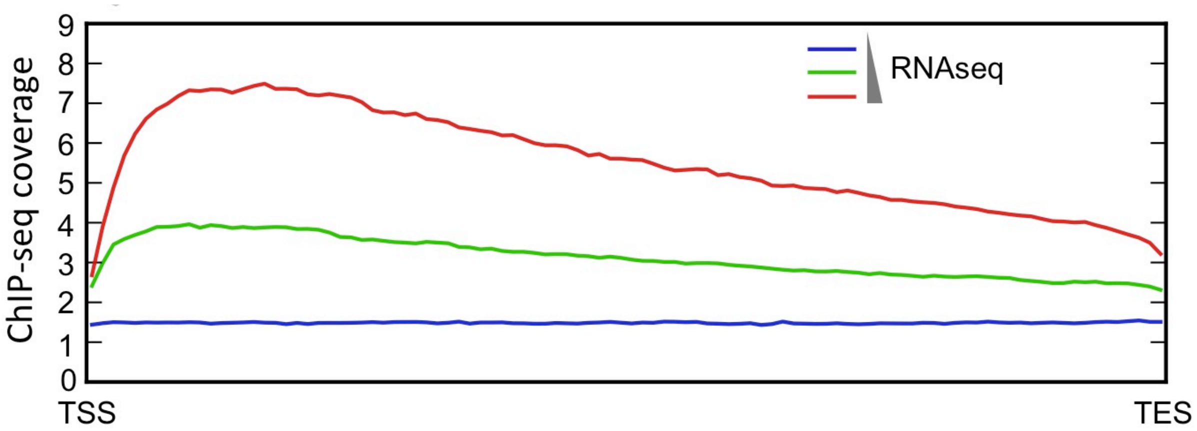

Supplementary Figure 1. **ChIP-seq profile of H2BK120ub in HeLa cells.** H2BK120ub is localized within gene bodies and is correlated with active transcription. H2BK120ub ChIP-seq coverage was scaled to a 1kb distance from the TSS to the TES. Genes were divided into three groups, based on their RNA-seq levels (quantified as reads per kilobase per million reads; RPKM). Data processed as described in Materials and Methods (data source listed in Supplementary Table 3).

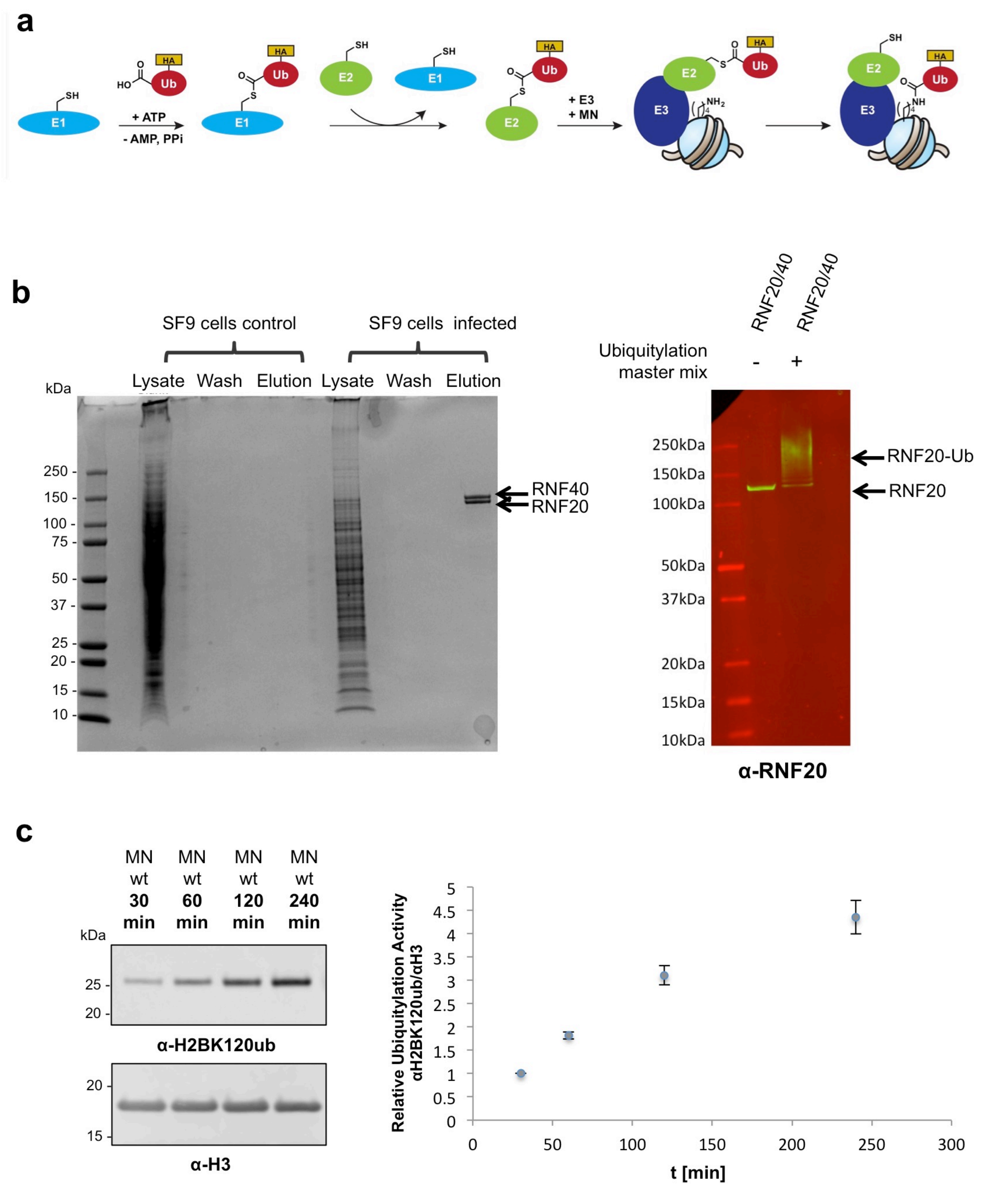

Supplementary Figure 2. *In vitro* ubiquitylation of H2BK120. (**a**) Schematic presentation of the RING-type ubiquitylation mechanism illustrated here using an HA-tagged ubiquitin as employed in the library-based screen. (**b**) Left: RNF20/40 expression and purification in SF9 cells analyzed by SDS-PAGE (stained with Coomassie Brilliant Blue). Right: α-RNF20 immunoblot with and without ubiquitylation master mix containing hE1 and UBE2A. Autoubiquitylation of functional RNF20/40 is observed in the presence of functional UBE2A. (**c**) *In vitro* ubiquitylation time course using MN substrates and native, unlabeled ubiquitin. Normalized H2BK120ub levels (αH2BK120ub/H3α) are plotted relative to the 30 min time point. Data are mean ± s.e.m. (n = 3). Full WB is shown in Supplementary Fig. 16a.

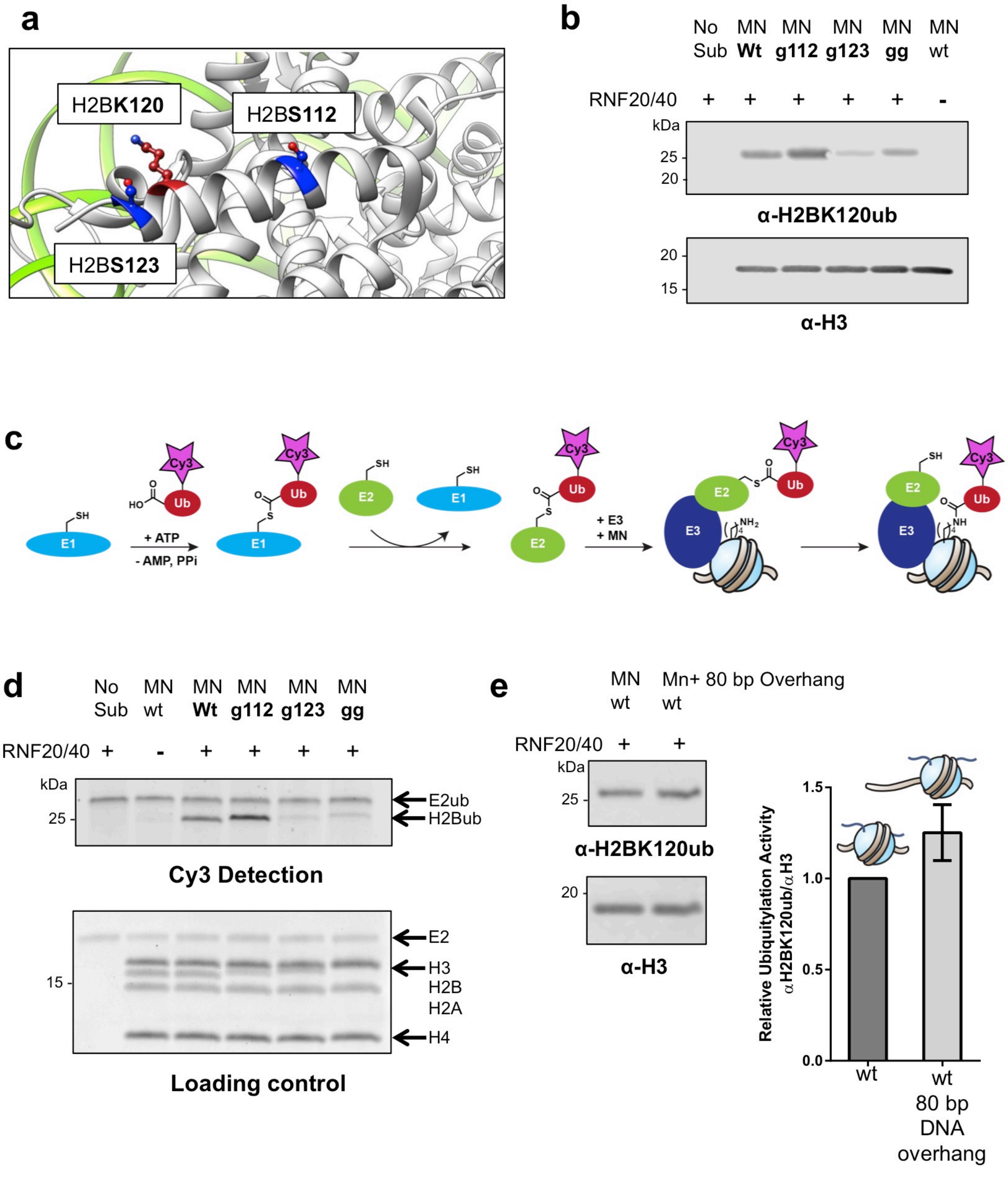

Supplementary Figure 3. Crosstalk between H2BK120ub and GlcNAcylation at the C-terminal helix of histone H2B. (**a**) Structure (PDB 1KX5) of the C-terminal helix of H2B within the nucleosome. GlcNAcylation sites are highlighted in blue and H2B K120 highlighted in red. Nucleosomal DNA is highlighted in green. (**b**) *In vitro* ubiquitylation of wt and GlcNAcylated mononucleosomes (MNs) (g112: H2BS112GlcNAc; g123: H2BS123GlcNAc; gg: H2BS112GlcNAc_S123GlcNAc) analyzed by western blotting. (**c**) Schematic presentation of the antibody-free Cy3-based *in vitro* ubiquitylation assay for a RING type E3 ligase. (**d**) *In vitro* ubiquitylation using Cy3-labeled ubiquitin and MNs as described in panel B. Cy3-readout by in-gel fluorescence and the corresponding protein stain used as loading control (SYPRO Ruby stain). (**e**) *In vitro* ubiquitylation of mononucleosomes with a minimal DNA overhang and mononucleosomes containing an asymmetrical 80 bp DNA overhang. Left: Representative western blot analysis after *in vitro* ubiquitylation. Right: Quantification of the immunoblotting data. Normalized H2BK120ub levels (αH2BK120ub/αH3) are plotted relative to wt-MNs. Data are mean ± s.e.m. (n = 3). Full gel images are presented in Supplementary Fig. 16b/c/d.

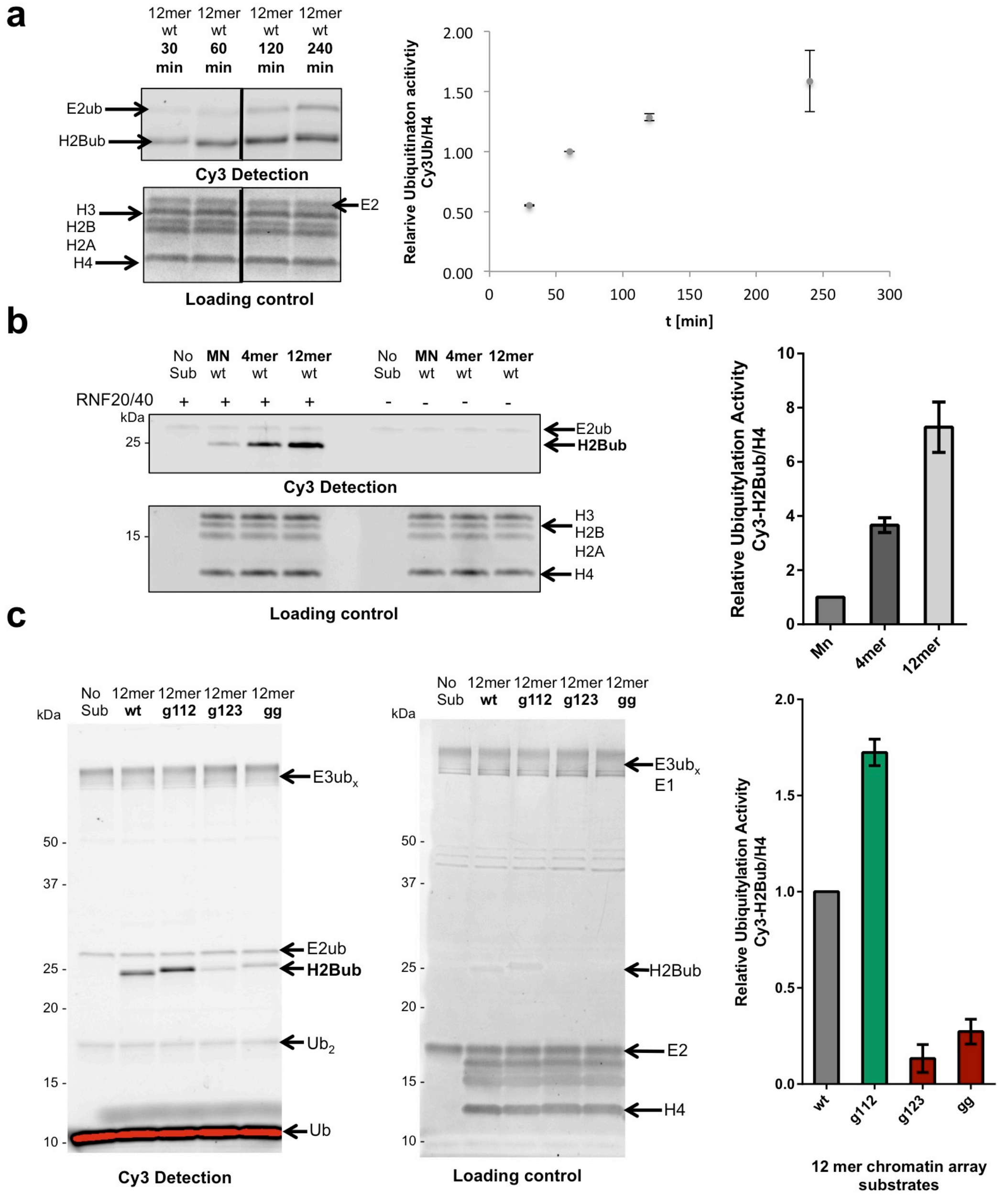

Supplementary Figure 4. *In vitro* ubiquitylation of 12mer chromatin arrays. (**a**) *In vitro* ubiquitylation time course using wt-12mer chromatin arrays and Cy3-labeled ubiquitin. Left: Representative SDS-PAGE after *in vitro* ubiquitylation (Cy3 fluorescence detection and SYPRO Ruby protein stain as loading control). The image was cropped for illustration purposes between lane 60 min and lane 120 min. Right: Quantification of the relative ubiquitylation levels by in-gel fluorescence. Normalized ubiquitylation levels (Cy3-H2Bub/H4) are plotted relative to the 60 min time point (n = 2). (**b**) *In vitro* ubiquitylation of mono- and oligonucleosomes. Left: Representative Cy3 readout and loading control. Right: Quantification of ubiquitylation activities (Cy3-H2Bub/H4) of wt mononucleosomes, 4mer and 12mer chromatin arrays. Ubiquitylation activities are normalized to MNs (n = 3). (**c**) *In vitro* ubiquitylation of wt- and GlcNAcylated-12mer chromatin arrays (g112: H2BS112GlcNAc; g123: H2BS123GlcNAc; gg: H2BS112GlcNAc_S123GlcNAc) using Cy3-labeled ubiquitin. Left: Representative Cy3 readout and loading control. Right: Quantification of ubiquitylation activities (Cy3-H2Bub/H4). Normalized ubiquitylation levels (Cy3-H2Bub/H4) are plotted relative to wt(n = 4). All data are mean ± s.e.m. Full gel images are given in Supplementary Fig. 17.

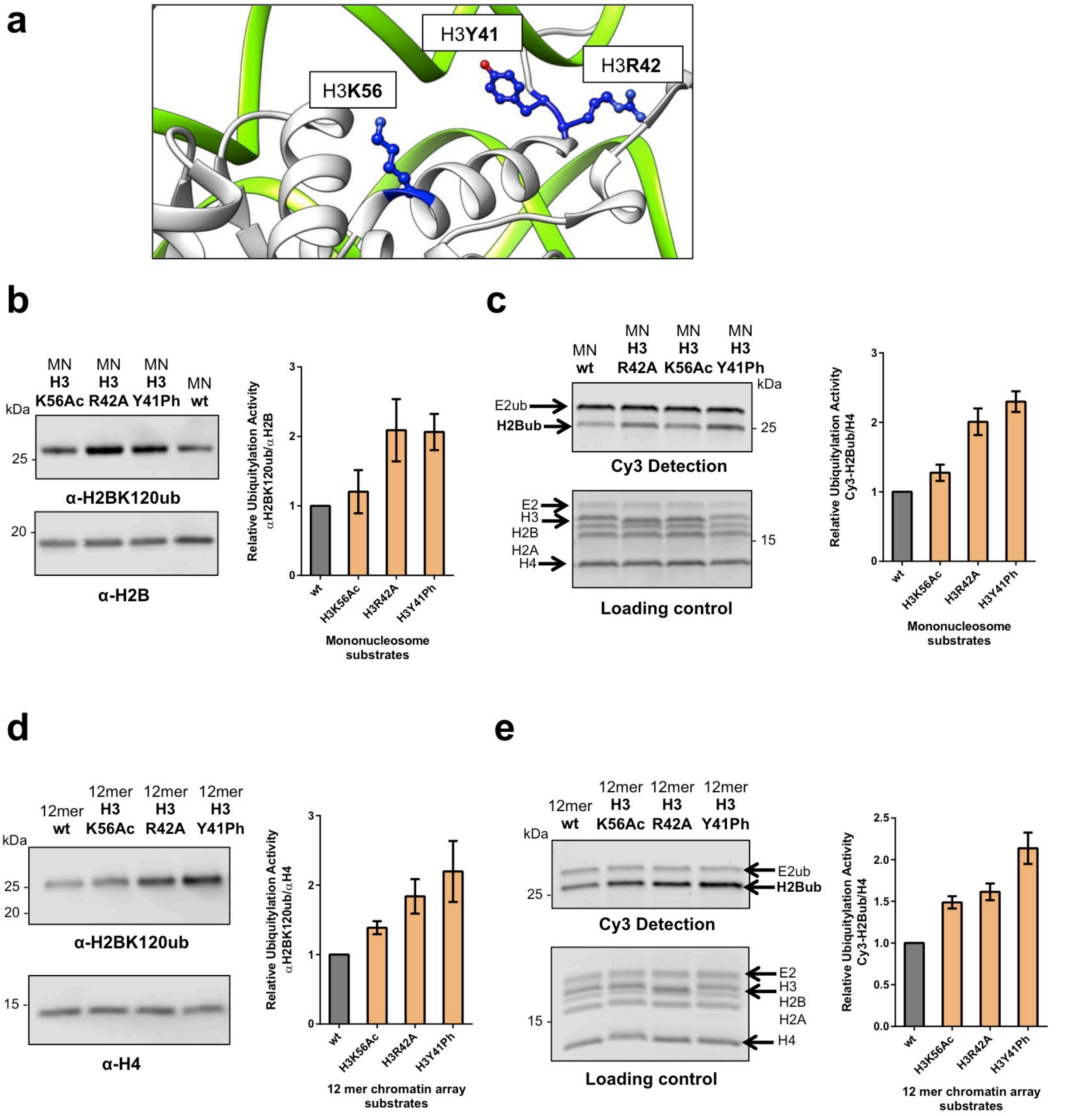

Supplementary Figure 5. Crosstalk between H2BK120ub and modification/mutants at the DNA entry/exit site. (**a**) Structure of the DNA entry/exit site of the nucleosome (PDB 1KX5). Positions H3Y41, H3R42A and H356 and their respective side chains are highlighted in blue. Nucleosomal DNA is highlighted in green. (**b,c**) *In vitro* ubiquitylation of wt- and modified MNs. (**b**) Left: Western blot analysis of *in vitro* ubiquitylation. Right: Quantification of immunoblot data (αH2BK120ub/αH2B) relative to wt (n = 3). (**c**) Left: In-gel fluorescence and protein stain (SYPRO Ruby) as loading control. Right: Quantification of ubiquitylation levels (Cy3-H2Bub/H4) relative to wt (n = 4). (**d,e**) *In vitro* ubiquitylation of wt- and modified 12mer chromatin arrays. (**d**) Left: Western blot analysis of *in vitro* ubiquitylation. Right: Quantification of immunoblot data (αH2BK120ub/αH4) relative to wt (n = 4). (**e**) Left: In-gel fluorescence and protein stain (SYPRO Ruby) as loading control. Right: Quantification of ubiquitylation levels (Cy3-H2Bub/H4) relative to wt (n = 3). All data are mean ± s.e.m. Full images are shown in Supplementary Fig. 18.

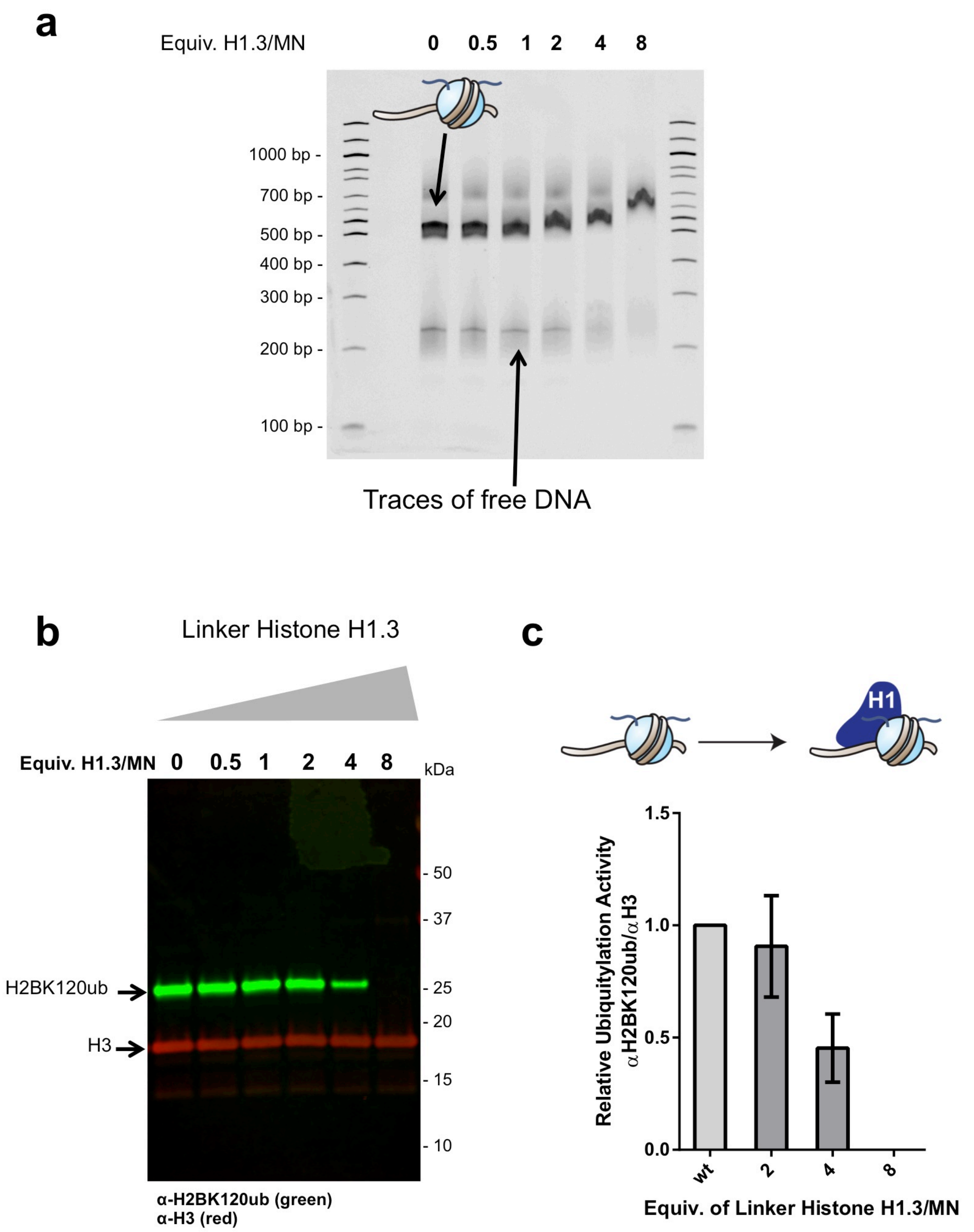

Supplementary Figure 6. Addition of linker histone H1.3 inhibits ubiquitylation of H2BK120 *in vitro*. (**a**) Native gel electrophoresis of MNs with a 80bp DNA overhang and different amounts of linker histone H1.3 (SYBR Gold nucleic acid stain). The gel shift indicates binding of linker histone H1.3. (**b,c**) *In vitro* ubiquitylation of MNs with a 80bp DNA overhang in presence of linker histone H1.3. (**b**) Western blot analysis of the *in vitro* ubiquitylation assay. (**c**) Quantification of immunoblot data (αH2BK120ub/αH3) relative to wt. Data are mean ± s.e.m. (n = 3). *In vitro* ubiquitylation was performed using standard conditions as described in the methods section (substrate concentration: 1μM of nucleosomal H2B corresponds to 0.5 μM mononucleosomes).

a

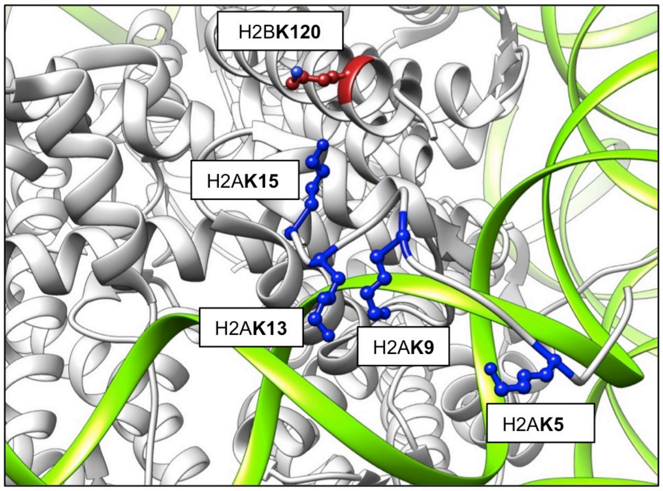

b

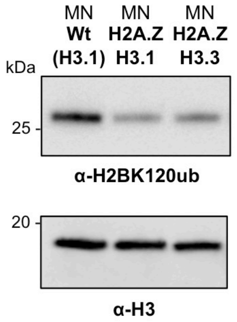

c

Wt-H2A and H2A.Z

Human H2A type 2A

```
        10         20         30         40         50         60
SGRGKQGGKA RAKAKSRSSR AGLQFPVGRV HRLLRKGNYA ERVGAGAPVY MAAVLEYLTA

        70         80         90        100        110        120
EILELAGNAA RDNKKTRIIP RHLQLAIRND EELNKLLGKV TIAQGGVLPN IQAVLLPKKT

ESHHKAKGK
```

Human H2A.Z

```
        10         20         30         40         50         60
AGGKAGKDSG KAKTKAVSRS QRAGLQFPVG RIHRHLKSRT TSHGRVGATA AVYSAAILEY

        70         80         90        100        110        120
LTAEVLELAG NASKDLKVKR ITPRHLQLAI RGDEELDSLI KATIAGGGVI PHIHKSLIGK

KGQQKTV
```

H2A_K15 Mutants

Human H2A type 2A K15V

```
        10         20         30         40         50         60
SGRGKQGGKA RAKAVSRSSR AGLQFPVGRV HRLLRKGNYA ERVGAGAPVY MAAVLEYLTA

        70         80         90        100        110        120
EILELAGNAA RDNKKTRIIP RHLQLAIRND EELNKLLGKV TIAQGGVLPN IQAVLLPKKT

ESHHKAKGK
```

Human H2A type 2A K15Q

```
        10         20         30         40         50         60
SGRGKQGGKA RAKAQSRSSR AGLQFPVGRV HRLLRKGNYA ERVGAGAPVY MAAVLEYLTA

        70         80         90        100        110        120
EILELAGNAA RDNKKTRIIP RHLQLAIRND EELNKLLGKV TIAQGGVLPN IQAVLLPKKT

ESHHKAKGK
```

H2A_Tail Acetylation Mimic (poly Q, pQ)

Human H2A type 2A K5,9,13,15Q

```
        10         20         30         40         50         60
SGRGQQGGQA RAQAQSRSSR AGLQFPVGRV HRLLRKGNYA ERVGAGAPVY MAAVLEYLTA

        70         80         90        100        110        120
EILELAGNAA RDNKKTRIIP RHLQLAIRND EELNKLLGKV TIAQGGVLPN IQAVLLPKKT

ESHHKAKGK
```

Tail Swap (TS) chimeras

Human H2A type 2A tail swap (H2A_TS)

```
        10         20         30         40         50         60
AGGKAGKDSG KAKTKAVSRS QRAGLQFPVG RVHRLLRKGN YAERVGAGAP VYMAAVLEYL

        70         80         90        100        110        120
TAEILELAGN AARDNKKTRI IPRHLQLAIR NDEELNKLLG KVTIAQGGVL PNIQAVLLPK

       130
KTESHHKAKG K
```

Human H2A.Z tail swap (H2A.Z_TS)

```
        10         20         30         40         50         60
SGRGKQGGKA RAKAKSRSSR AGLQFPVGRI HRHLKSRTTS HGRVGATAAV YSAAILEYLT

        70         80         90        100        110        120
AEVLELAGNA SKDLKVKRIT PRHLQLAIRG DEELDSLIKA TIAGGGVIPH IHKSLIGKKG

QQKTV
```

Supplementary Figure 7. Crosstalk between H2BK120ub and the N-terminal tail of H2A. (**a**) Structure of the H2A tail region within the nucleosome (PDB 1KX5). Positions H2AK15, H2AK13, H2AK9 and H2AK5 and the respective side chains are highlighted in blue. Nucleosomal DNA is highlighted in green. (**b**) *In vitro* ubiquitylation of MNs containing histone variant H2A.Z paired with H3.1 and H3.3. Western Blot analysis of *in vitro* ubiquitylation reactions. (**c**) Full amino acid sequences of H2A/H2A.Z mutants and chimeras employed in this study. Introduced changes to the canonical H2A/H2A.Z amino acid sequences are highlighted in red. Full WB is presented in Supplementary Fig. 19a.

Mus Musculus (Mouse)

UniProt ID:

```
                                                K5  K9  K13 K15*
Q8CGP5    H2A1F_MOUSE     1    --MSGRGKQGGKARAKAKTRSSRAGLQFPVGRVHRLLRKGN-YSERVGAGAPVYLAAVLE    57
Q8CGP7    H2A1K_MOUSE     1    --MSGRGKQGGKARAKAKTRSSRAGLQFPVGRVHRLLRKGN-YSERVGAGAPVYLAAVLE    57
C0HKE9    H2A1P_MOUSE     1    --MSGRGKQGGKARAKAKTRSSRAGLQFPVGRVHRLLRKGN-YSERVGAGAPVYLAAVLE    57
Q6GSS7    H2A2A_MOUSE     1    --MSGRGKQGGKARAKAKSRSSRAGLQFPVGRVHRLLRKGN-YAERVGAGAPVYMAAVLE    57
P27661    H2AX_MOUSE      1    --MSGRGKTGGKARAKAKSRSSRAGLQFPVGRVHRLLRKGH-YAERVGAGAPVYLAAVLE    57
C0HKE2    H2A1C_MOUSE     1    --MSGRGKQGGKARAKAKTRSSRAGLQFPVGRVHRLLRKGN-YSERVGAGAPVYLAAVLE    57
C0HKE1    H2A1B_MOUSE     1    --MSGRGKQGGKARAKAKTRSSRAGLQFPVGRVHRLLRKGN-YSERVGAGAPVYLAAVLE    57
Q64522    H2A2B_MOUSE     1    --MSGRGKQGGKARAKAKSRSSRAGLQFPVGRVHRLLRKGN-YAERVGAGAPVYMAAVLE    57
Q64523    H2A2C_MOUSE     1    --MSGRGKQGGKARAKAKSRSSRAGLQFPVGRVHRLLRKGN-YAERVGAGAPVYMAAVLE    57
C0HKE5    H2A1G_MOUSE     1    --MSGRGKQGGKARAKAKTRSSRAGLQFPVGRVHRLLRKGN-YSERVGAGAPVYLAAVLE    57
Q8CGP6    H2A1H_MOUSE     1    --MSGRGKQGGKARAKAKTRSSRAGLQFPVGRVHRLLRKGN-YSERVGAGAPVYLAAVLE    57
C0HKE8    H2A1O_MOUSE     1    --MSGRGKQGGKARAKAKTRSSRAGLQFPVGRVHRLLRKGN-YSERVGAGAPVYLAAVLE    57
Q8R1M2    H2AJ_MOUSE      1    --MSGRGKQGGKVRAKAKSRSSRAGLQFPVGRVHRLLRKGN-YAERVGAGAPVYLAAVLE    57
C0HKE4    H2A1E_MOUSE     1    --MSGRGKQGGKARAKAKTRSSRAGLQFPVGRVHRLLRKGN-YSERVGAGAPVYLAAVLE    57
C0HKE6    H2A1I_MOUSE     1    --MSGRGKQGGKARAKAKTRSSRAGLQFPVGRVHRLLRKGN-YSERVGAGAPVYLAAVLE    57
C0HKE7    H2A1N_MOUSE     1    --MSGRGKQGGKARAKAKTRSSRAGLQFPVGRVHRLLRKGN-YSERVGAGAPVYLAAVLE    57
Q8BFU2    H2A3_MOUSE      1    --MSGRGKQGGKARAKAKSRSSRAGLQFPVGRVHRLLRKGN-YSERVGAGAPVYLAAVLE    57
C0HKE3    H2A1D_MOUSE     1    --MSGRGKQGGKARAKAKTRSSRAGLQFPVGRVHRLLRKGN-YSERVGAGAPVYLAAVLE    57
P0C0S6    H2AZ_MOUSE      1    MAGGKAGKDSGKAKTKAVSRSQRAGLQFPVGRIHRHLKSRTTSHGRVGATAAVYSAAILE    60
Q3THW5    H2AV_MOUSE      1    MAGGKAGKDSGKAKAKAVSRSQRAGLQFPVGRIHRHLKTRTTSHGRVGATAAVYSAAILE    60
```

Xenapus laevis (African clawed frog)

```
Q6GM86    H2AX_XENLA      1    --MSGRGKAVSKTRAKAKTRSSRAGLQFPVGRVHRLLRKGN-YAHRVGAGAPVYLAAVLE    57
P06897    H2A1_XENLA      1    --MSGRGKQGGKTRAKAKTRSSRAGLQFPVGRVHRLLRKGN-YAERVGAGAPVYLAAVLE    57
P06898    H2A2_XENLA      1    --MSGRGKQGGKTRAKSKTRSSRAGLQFPVGRVHRLLRKGN-YAERVGAGAPVYLAAVLE    57
Q6GM74    H2AV_XENLA      1    MAGGKAGKDSGKAKAKAVSRSQRAGLQFPVGRIHRHLKTRTTSHGRVGATAAVYSAAILE    60
P70094    H2AZL_XENLA     1    MAGGKAGKDTGKAKATSITRSSRAGLQFPVGRIHRLLKNRTTSHGRVGGTAAVYTAAILE    60
```

Gallus gallus (Chicken)

```
P35062    H2A3_CHICK      1    --MSGRGKQGGKARAKAKSRSSRAGLQFPVGRVHRLLRKGN-YAERVGAGAPVYLAAVLE    57
P70082    H2AJ_CHICK      1    --MSGRGKQGGKVRAKAKSRSSRAGLQFPVGRVHRLLRKGN-YAERVGAGAPVYMAAVLE    57
P02263    H2A4_CHICK      1    --MSGRGKQGGKARAKAKSRSSRAGLQFPVGRVHRLLRKGN-YAERVGAGAPVYLAAVLE    57
Q5ZMD6    H2AZ_CHICK      1    MAGGKAGKDSGKTKTKAVSRSQRAGLQFPVGRIHRHLKSRTTSHGRVGATAAVYSAAILE    60
P02272    H2AV_CHICK      1    MAGGKAGKDSGKAKAKAVSRSQRAGLQFPVGRIHRHLKTRTTSHGRVGATAAVYSAAILE    60
```

Drosophila melanogaster (Fruit fly)

```
P84051    H2A_DROME       1    --MSGRGK-GGKVKGKAKSRSNRAGLQFPVGRIHRLLRKGN-YAERVGAGAPVYLAAVME    56
P08985    H2AV_DROME      1    MAGGKAGKDSGKAKAKAVSRSARAGLQFPVGRIHRHLKSRTTSHGRVGATAAVYSAAILE    60
```

Caenorhabditis elegans (Roundworm)

```
P09588    H2A_CAEEL       1   -MSGRGKGGKA--KTGGKAKSRSSRAGLQFPVGRLHRILRKGN-YAQRVGAGAPVYLAAVLE    58
Q27511    H2AV_CAEEL      1   MAGGKGKAGKDSGKSKSKVVSRSARAGLQFPVGRIHRFLKQRTTSSGRVGATAAVYSAAILE    62
```

Saccharomyces cerevisiae (Baker's yeast)

```
P04911    H2A1_YEAST    1   MS----G--GKGGKAGSAAKASQSRSAKAGLTFPVGRVHRLLRRGN-YAQRIGSGAPVYLTAVLE    58
P04912    H2A2_YEAST    1   MS----G--GKGGKAGSAAKASQSRSAKAGLTFPVGRVHRLLRRGN-YAQRIGSGAPVYLTAVLE    58
Q12692    H2AZ_YEAST    1   MSGKAHGGKGKSGAKDSGSLRSQSSSARAGLQFPVGRIKRYLKRHATGRTRVGSKAAIYLTAVLE    65
```

Supplementary Figure 8. Interplay between Lys-15 of H2A and Val-17 of H2A.Z (and H2A.V) is highly conserved in metazoans. Amino acid sequence of canonical H2A and H2A variants were aligned using the UniProt database (H2A variants H2A.bbd and macroH2A are not shown in the alignment). H2AZL_Xena (UniProt ID P70094) encodes for histone H2A.Z-like variant found in Xenopus lavis.

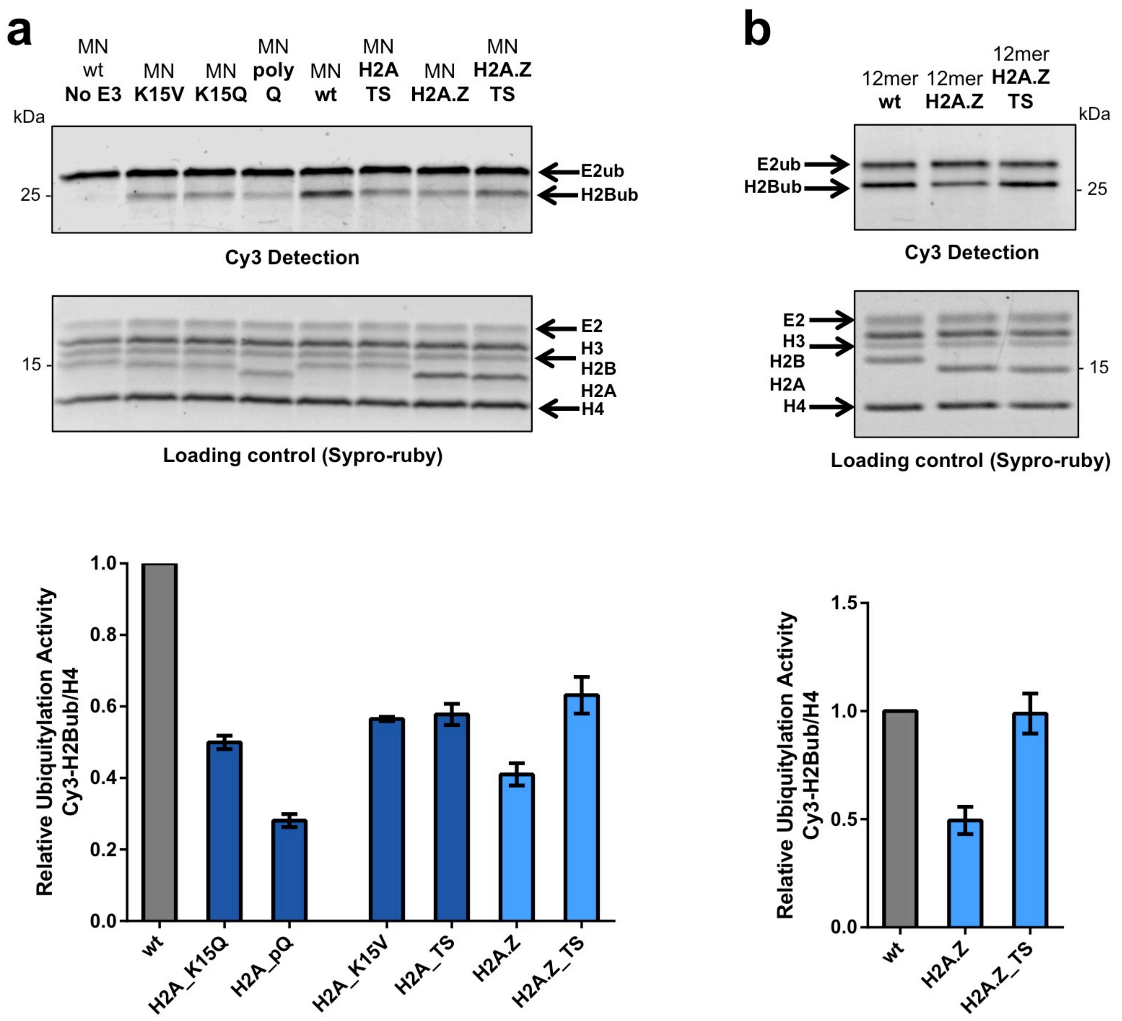

Supplementary Figure 9. Crosstalk between H2BK120ub and the N-terminal tail of H2A analyzed by *in vitro* ubiquitylation using Cy3-Ub. (**a**) *In vitro* ubiquitylation of mononucleosomes containing H2A/H2A.Z mutants using Cy3-Ub. Top: Cy3 readout and corresponding loading control (SYPRO Ruby stain). Bottom: Quantification of ubiquitylation activities (Cy3-H2Bub/H4) plotted relative to wt-MN (n = 4). (**b**) *In vitro* ubiquitylation of 12mer chromatin arrays containing H2A.Z and H2A.Z_TS measured by in-gel fluorescence. Top: Cy3 readout and corresponding loading control (SYPRO Ruby stain). Bottom: Quantification of ubiquitylation activities (Cy3-H2Bub/H4) plotted relative to wt-12mer chromatin arrays (n = 4). All data are mean ± s.e.m. Full gel images are shown in Supplementary Fig. 19b/c.

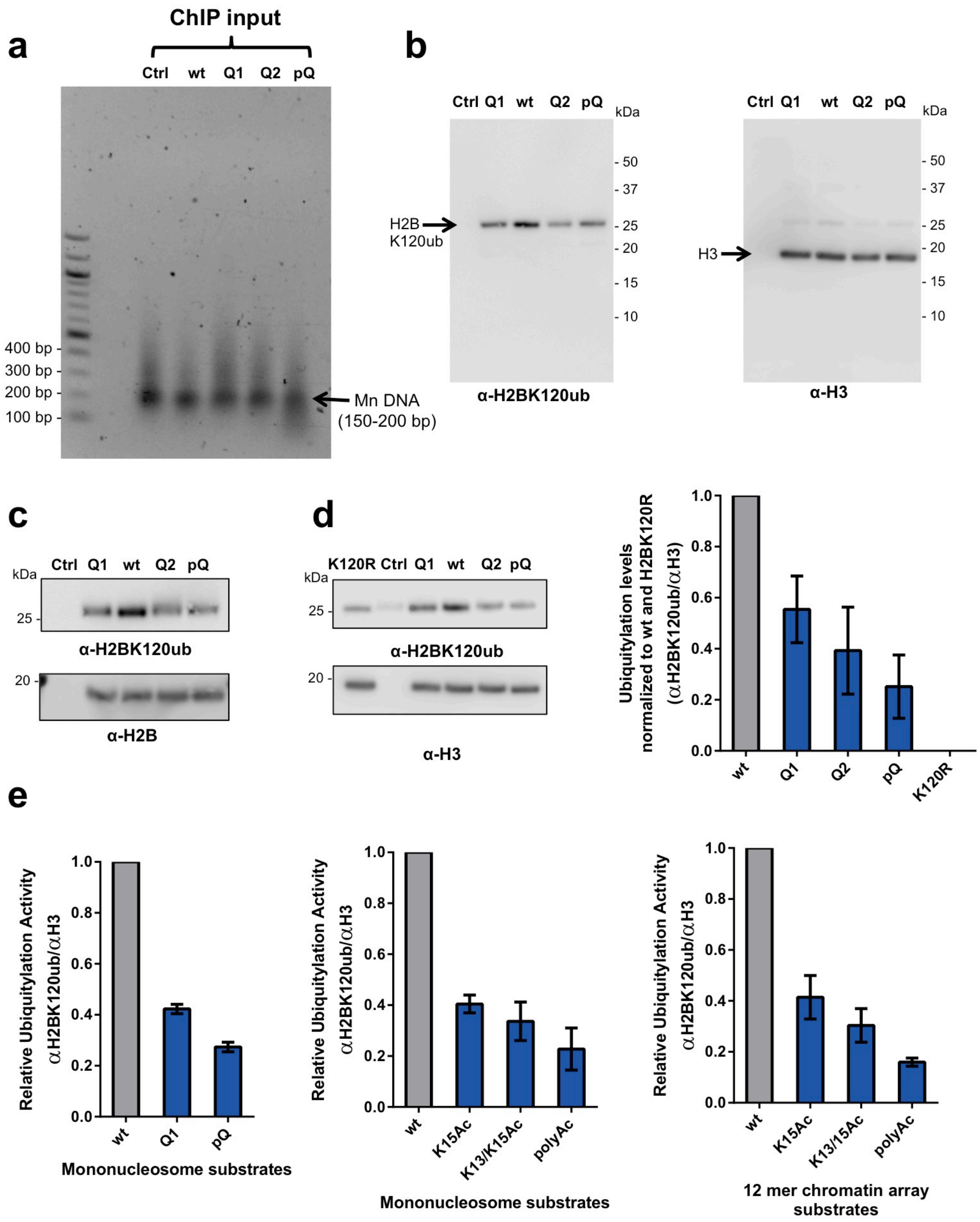

Supplementary Figure 10. Regulation of H2BK120ub by the H2A N-terminal tail in HEK293T cells. (**a**) Analysis of ChIP input (after MNase digestion and DNA isolation), showing that MNase digestion provided predominately MNs. (**b**) Full image of WB analysis, which was cropped for illustration purposes in Fig. 5a. (**c**) WB analysis of H2BK120ub levels after ChIP using H2B as loading control. (**d**) Left: Representative WB including a negative control: H2BK120R (K120R). Right: Quantification of H2BK120ub levels after ChIP in HEK 293T cells. Each data set of ubiquitylation levels (αH2BK120ub/αH3) was normalized to wt (100%) and H2BK120R (0%) followed by calculating the mean ± s.e.m. (n = 4). (**e**) Previously determined *in vitro* ubiquitylation activities for comparison: MN containing H2A K→ Q mutants (Fig. 3d), acetylated H2A (Fig. 3a) and 12mer chromatin arrays containing acetylated H2A (Fig. 3b). Full WBs are shown in Supplementary Fig. 20. Ctrl: Control (mock transfection); wt: H2A-FLAG; Q1: H2A_K15Q-FLAG; Q2: H2A_K13/15Q-FLAG; pQ: H2A_K5/9/13/15Q-FLAG).

Δ10H2A

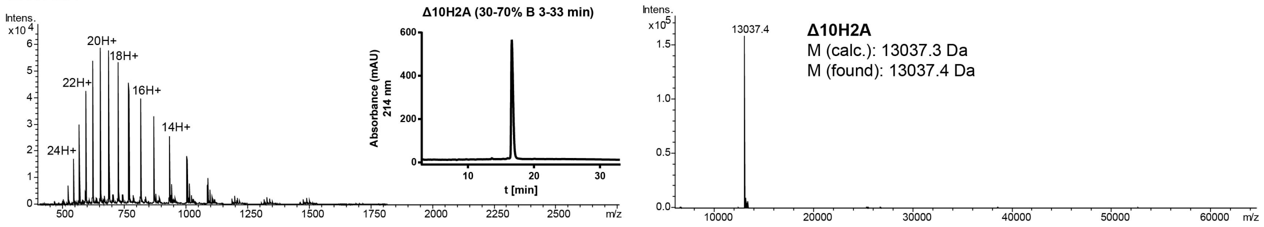

Δ15H2A

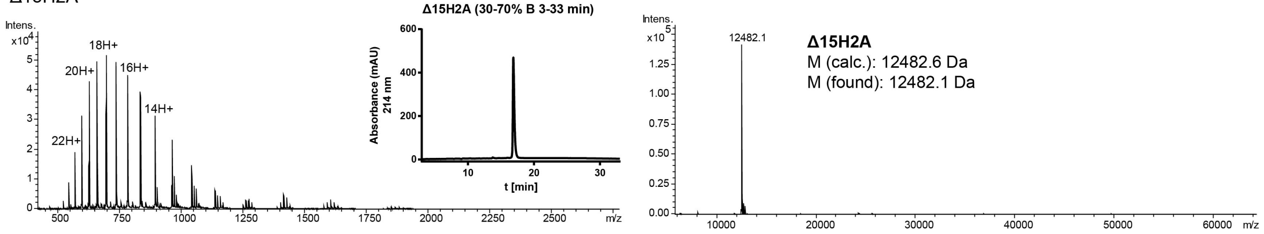

H2B_S112GlcNAc (g112)

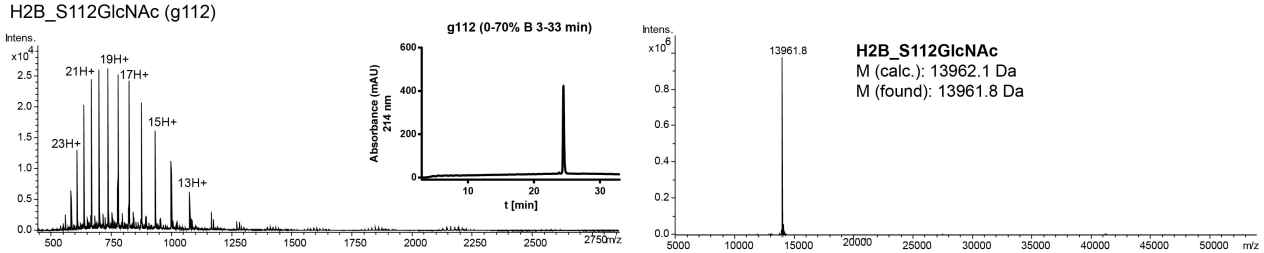

H2B_S123GlcNAc (g123)

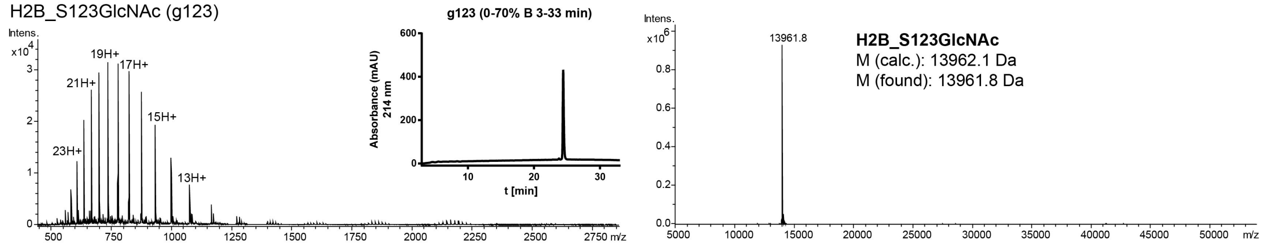

H2B_S112/S123GlcNAc (gg)

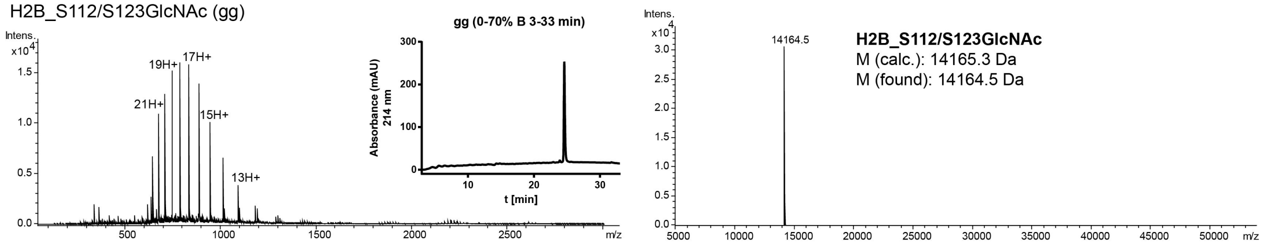

Cy3-Ub-OH

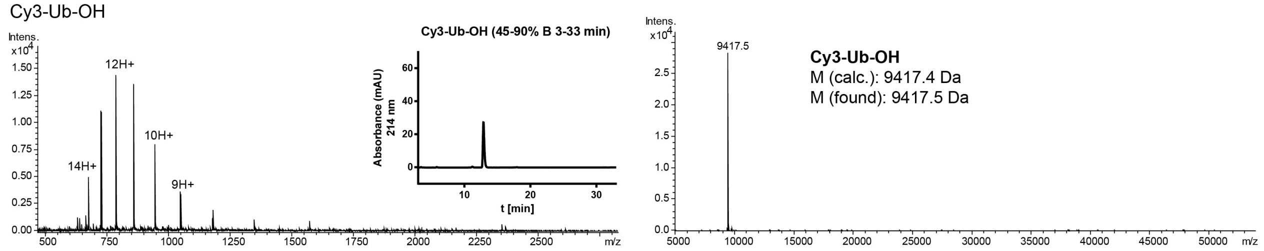

H2A_K5Ac

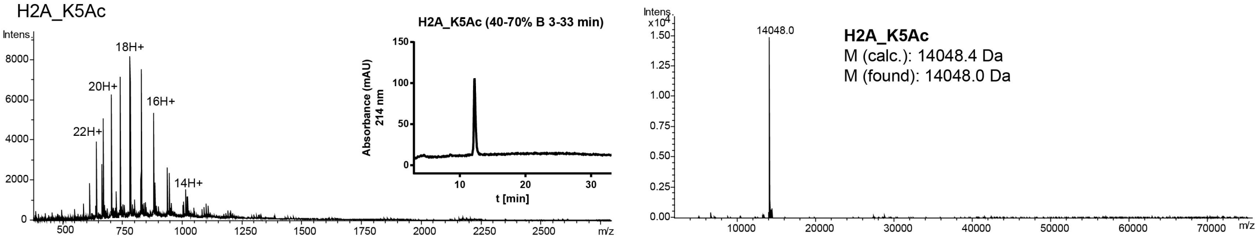

H2A_K9Ac

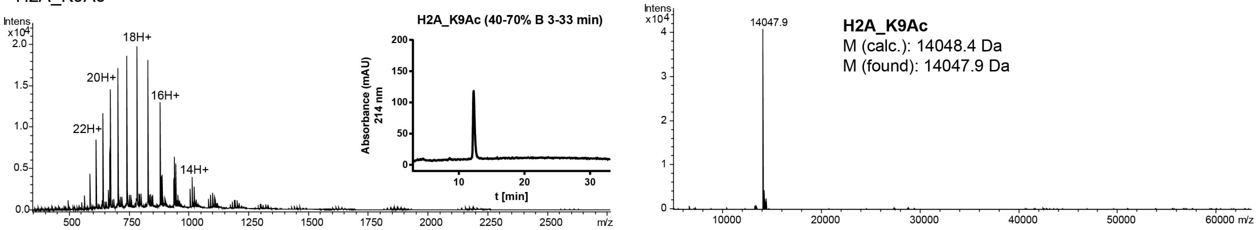

H2A_K13Ac

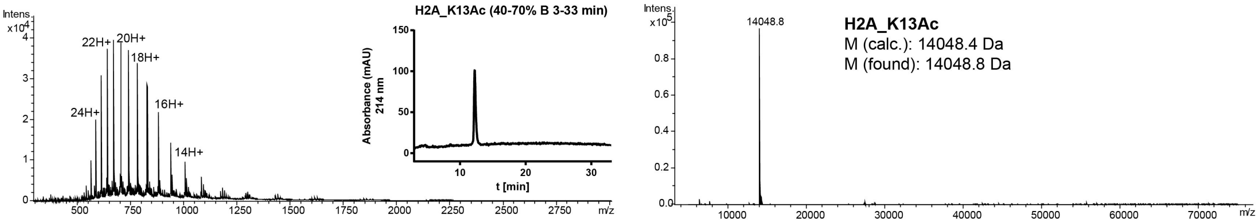

H2A_K15Ac

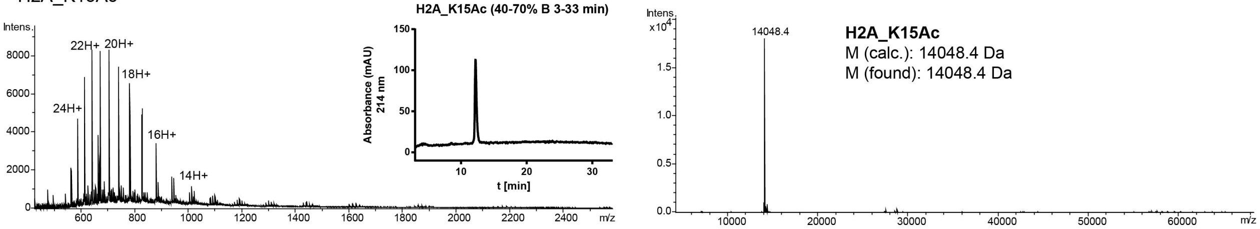

H2A_K13/15Ac (dAc)

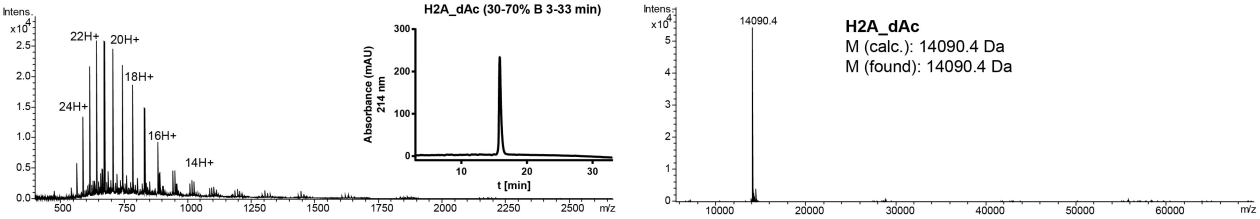

H2A_K5/9/13/15Ac (pAc)

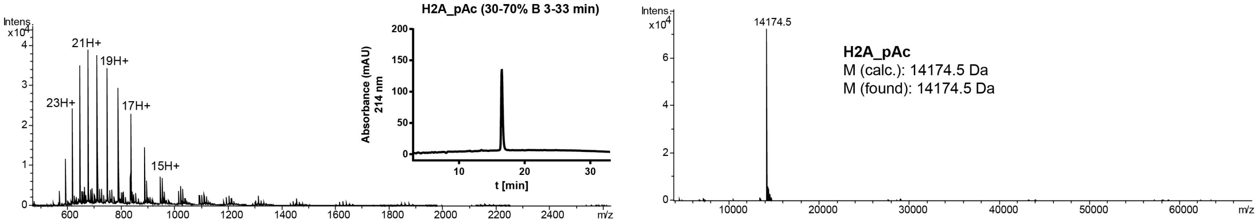

H2A_K15Q

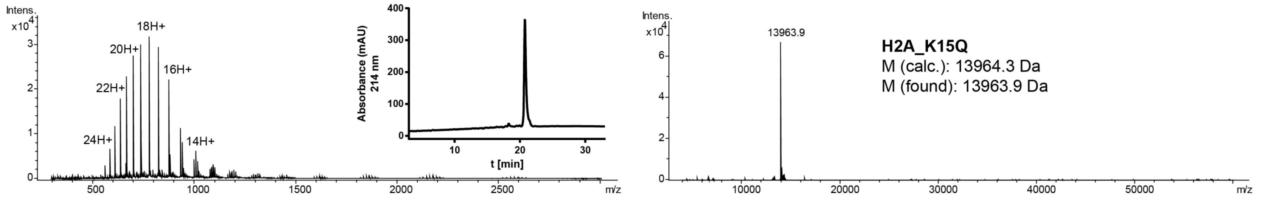

H2A_K15Q
M (calc.): 13964.3 Da
M (found): 13963.9 Da

H2A_K5/9/13/15Q (H2A_pQ)

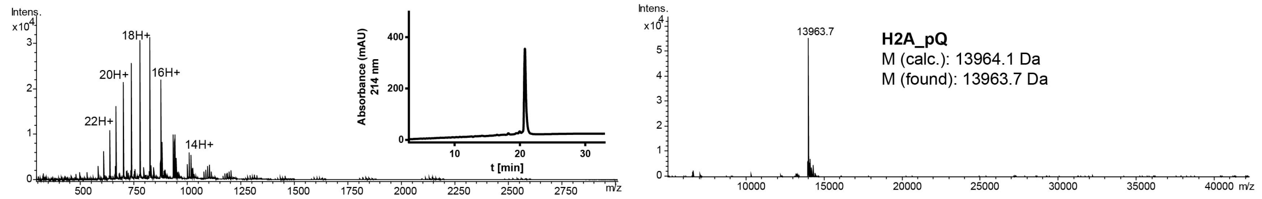

H2A_pQ
M (calc.): 13964.1 Da
M (found): 13963.7 Da

H2A_K15V

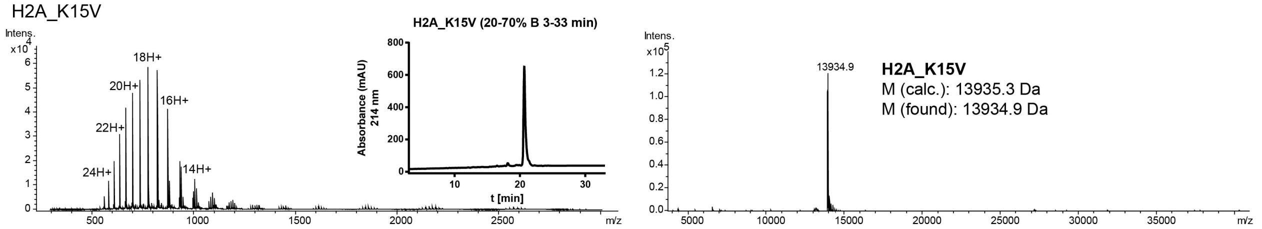

H2A_K15V
M (calc.): 13935.3 Da
M (found): 13934.9 Da

H2A_TS

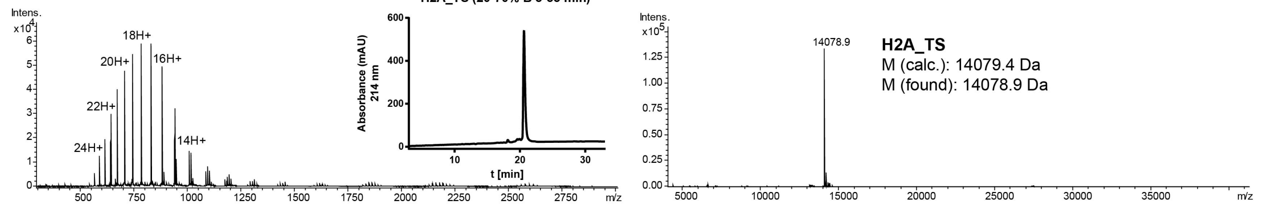

H2A_TS
M (calc.): 14079.4 Da
M (found): 14078.9 Da

H2A.Z_TS

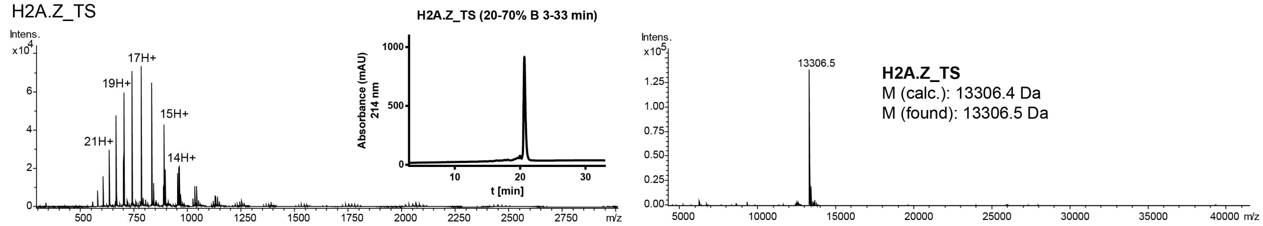

H2A.Z_TS
M (calc.): 13306.4 Da
M (found): 13306.5 Da

H3Y41Ph

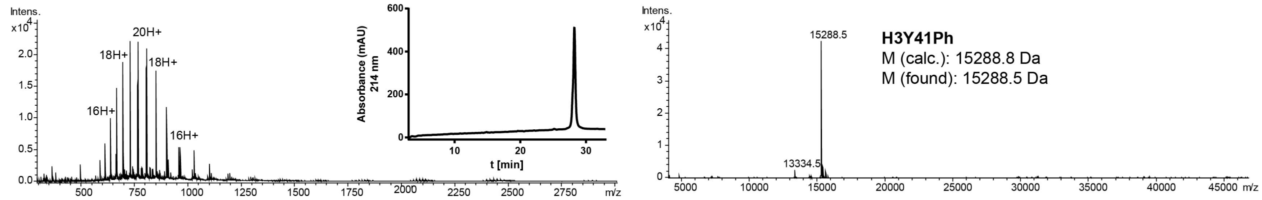

H3Y41Ph
M (calc.): 15288.8 Da
M (found): 15288.5 Da

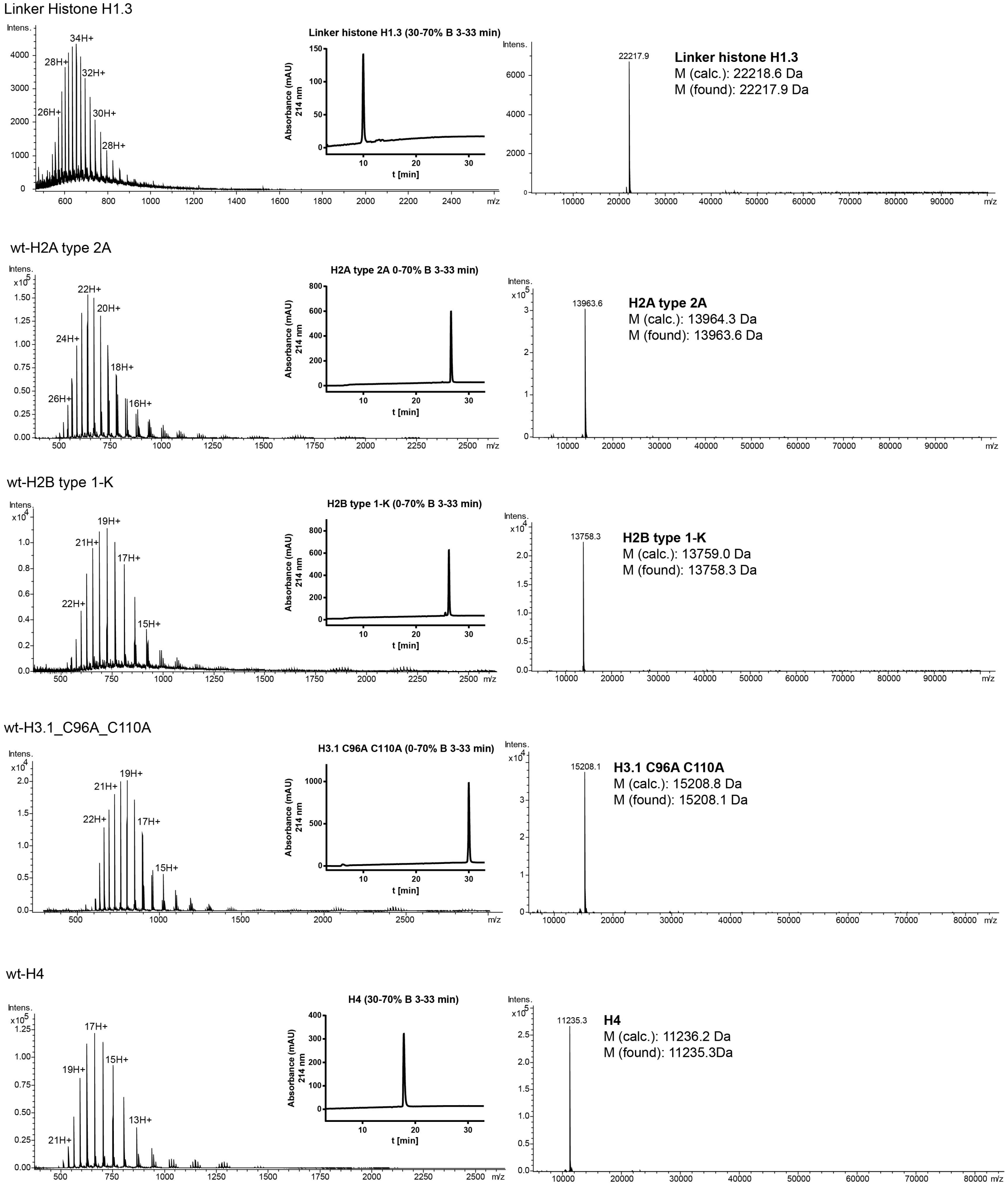

Supplementary Figure 11. Characterization of recombinant and semisynthetic proteins used in this study. Left: MS spectra of the indicated proteins and RP-HPLC chromatogram (solvent gradients are individually noted). Right: Deconvoluted MS-spectra and comparison of calculated and found MW. Serine modified with N-acetylglucosamine: SGlcNAc; Tail swap mutant: TS (see Supplementary Fig. 7c); Lysine acetylation: KAc; tyrosine phosphorylation: YPh.

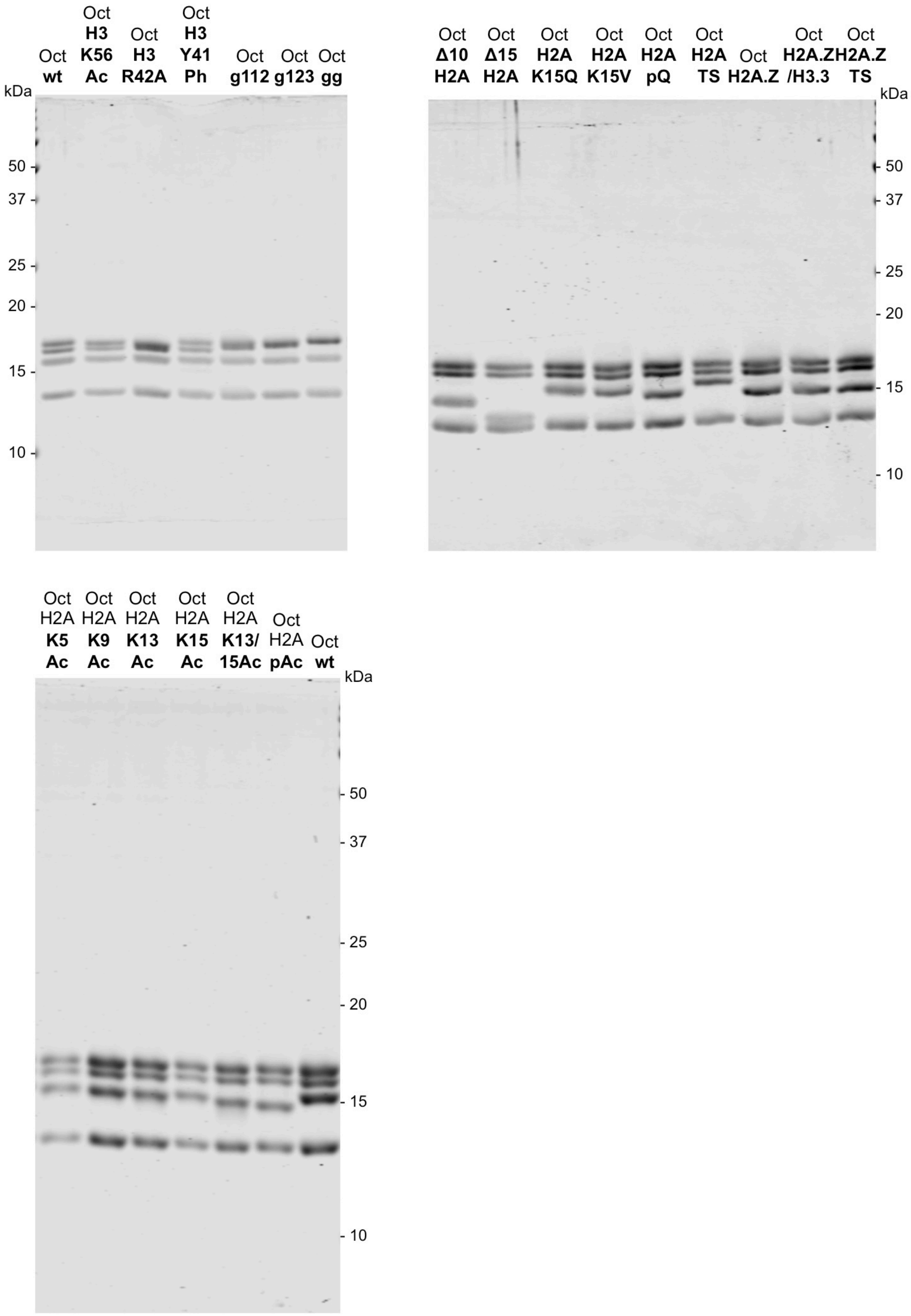

Supplementary Figure 12. Characterization of histone octamers. Individual histone octamers were analyzed by SDS-PAGE and stained with Coomassie Brilliant Blue.

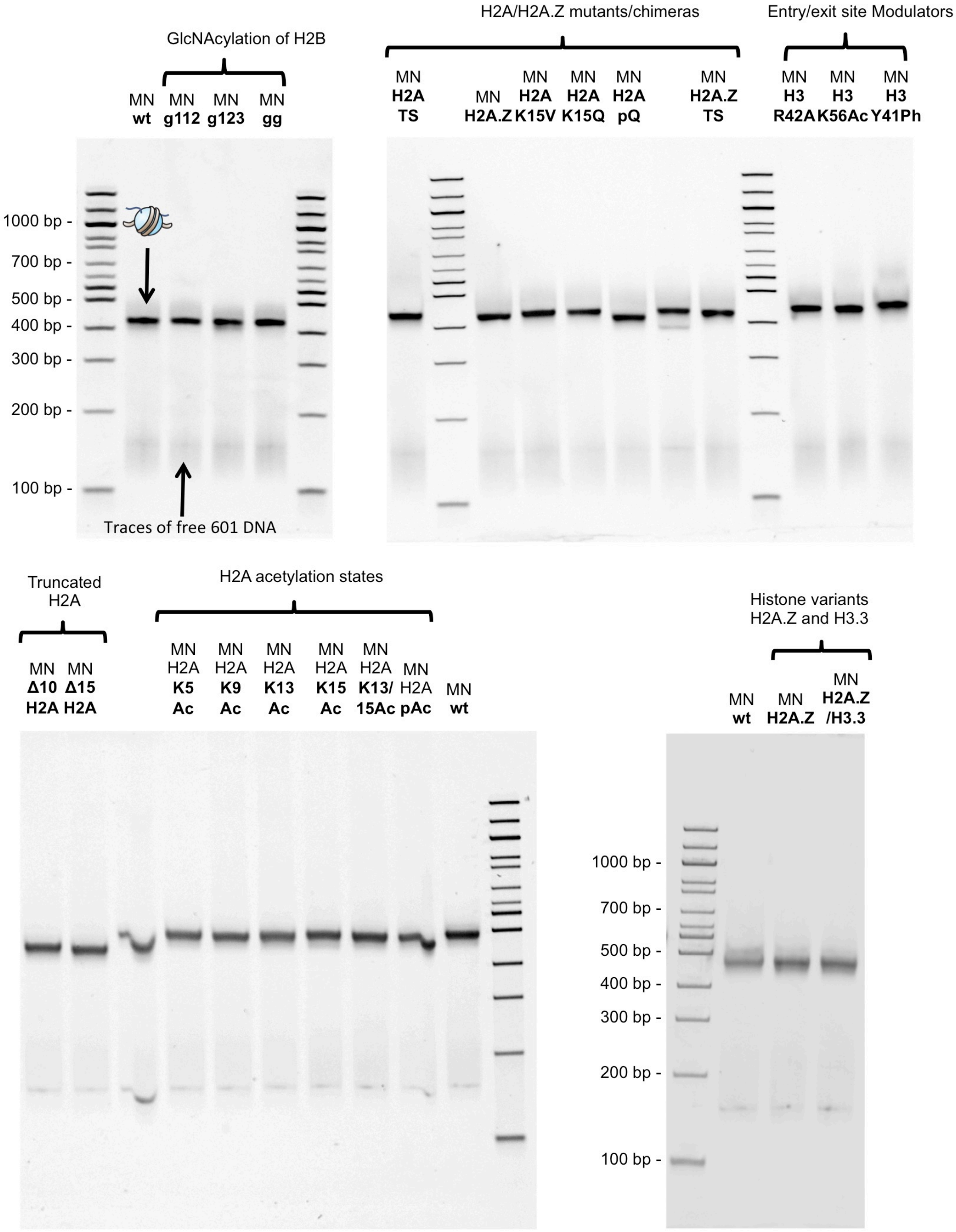

Supplementary Figure 13. Characterization of mononucleosome substrates. Individual MNs were analyzed by native gel electrophoresis and stained with SYBR Gold nucleic acid stain.

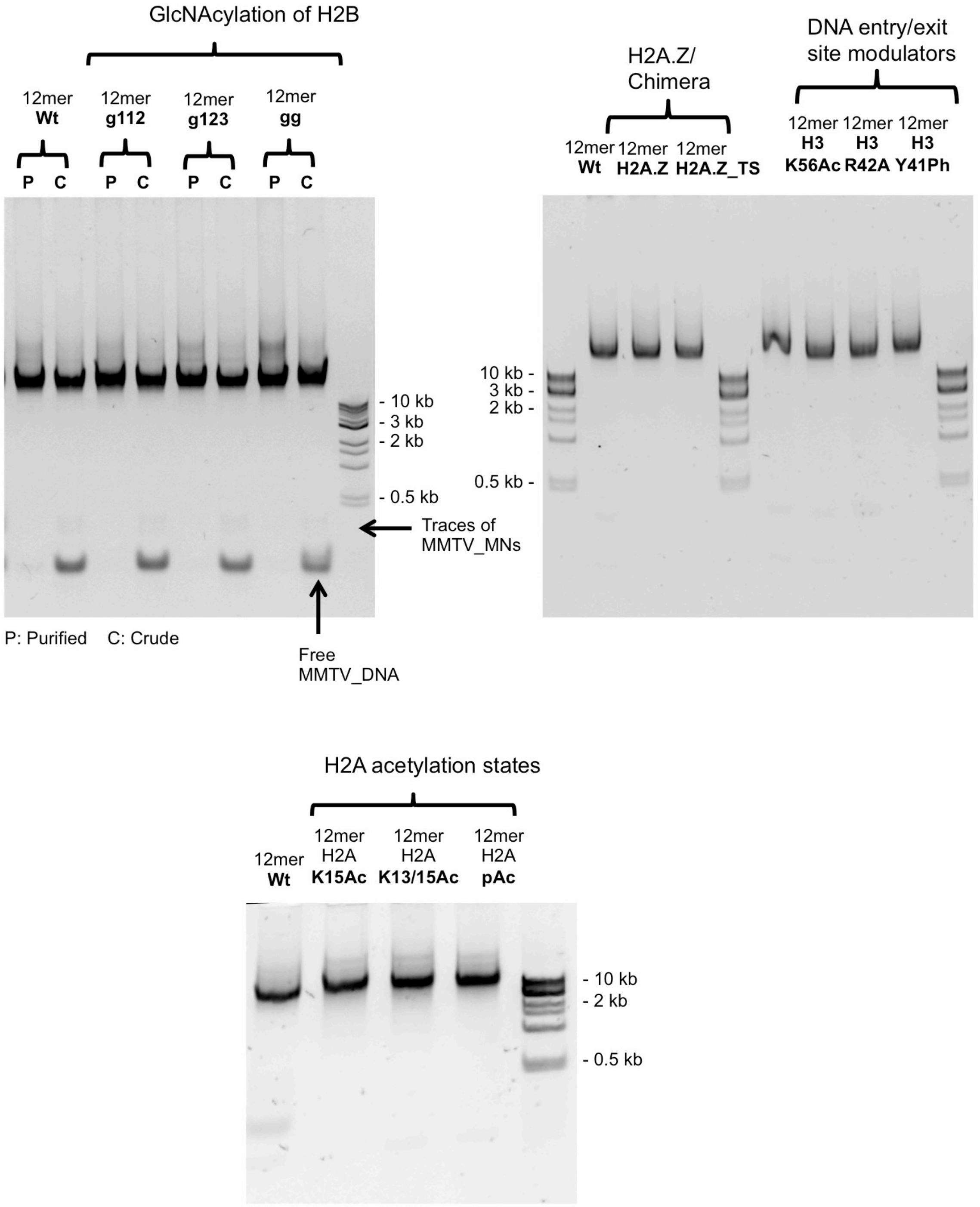

Supplementary Figure 14. Characterization of 12mer chromatin array substrates. Individual 12mer chromatin arrays were analyzed by native gel electrophoresis (APAGE gel) and stained with SYBR Gold nucleic acid stain. Crude assemblies and purified samples are shown for wt-, g112,- g123 -and gg-12mer chromatin arrays.

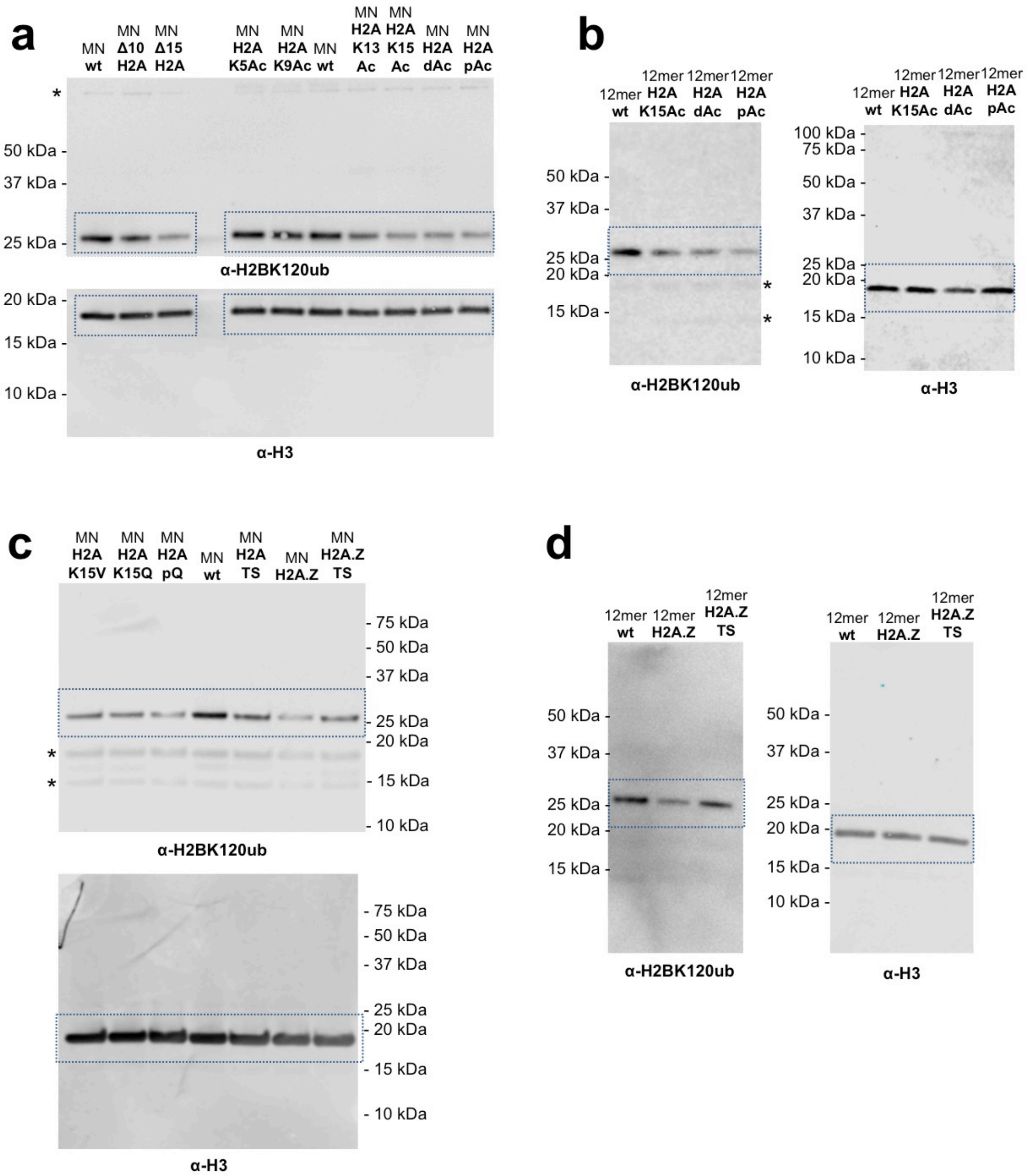

Supplementary Figure 15. Full images for data presented in Fig. 3. Blots were cropped for illustration purposes. (**a**) Full image for WB analysis presented in Fig. 3a. WB was physically cut before antibody incubation. (**b**) Full image for WB analysis presented in Fig. 3b. (**c**) Full image for WB analysis presented in Fig. 3d. The asterisks mark non-specific antibody binding. (**d**) Full image for WB analysis presented in Fig. 3e.

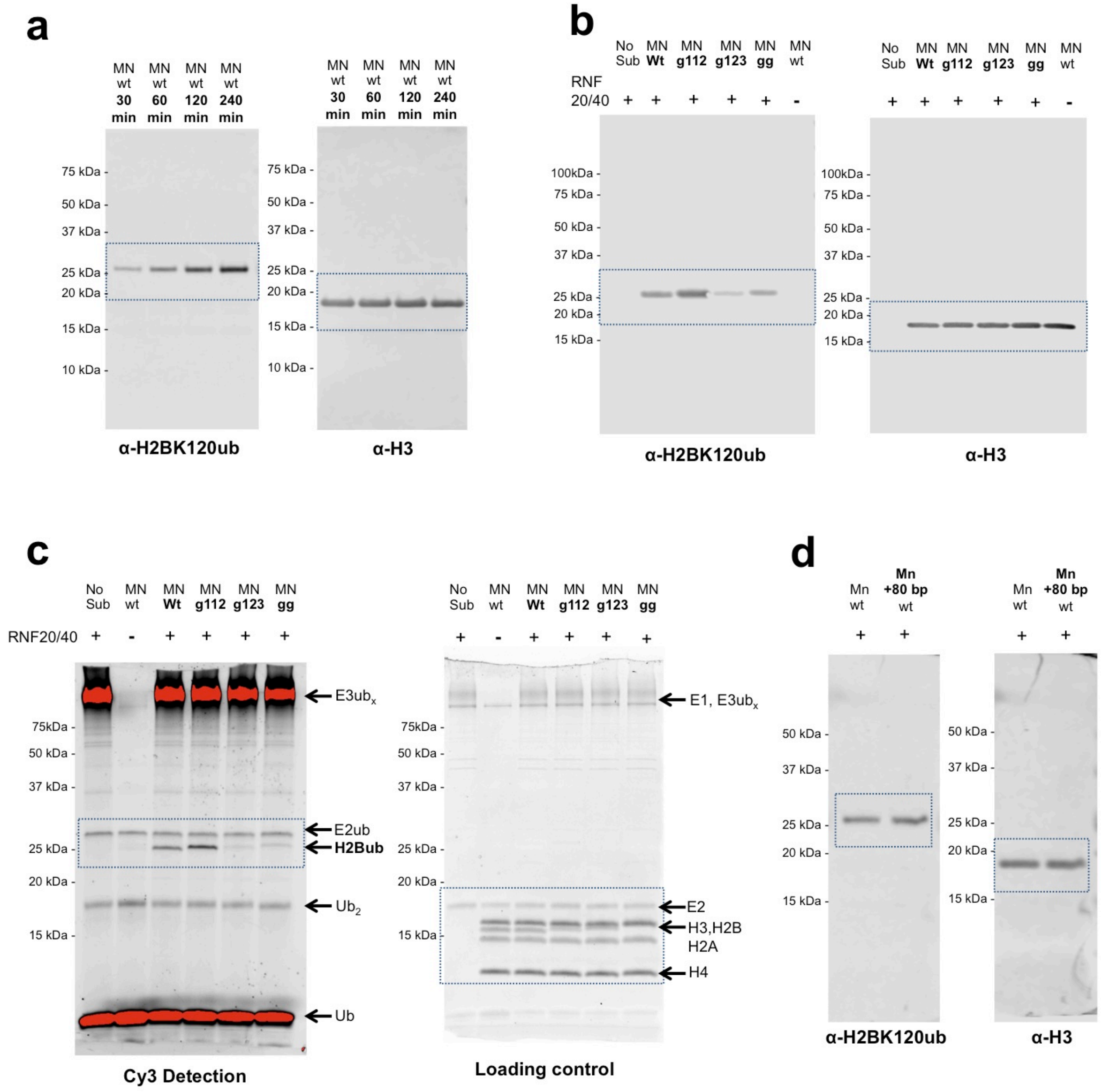

Supplementary Figure 16. Full images for data presented in Supplementary Fig 2/3. Images were cropped for illustration purposes. (**a**) Full image for WB analysis presented in Supplementary Fig. 2c. (**b**) Full image for WB analysis presented in Supplementary Fig. 3b. (**c**) Full image of Cy3 readout and loading control (SYPRO Ruby protein stain) presented in Supplementary Fig. 3d. (**d**) Full image for WB analysis presented in Supplementary Fig. 3e.

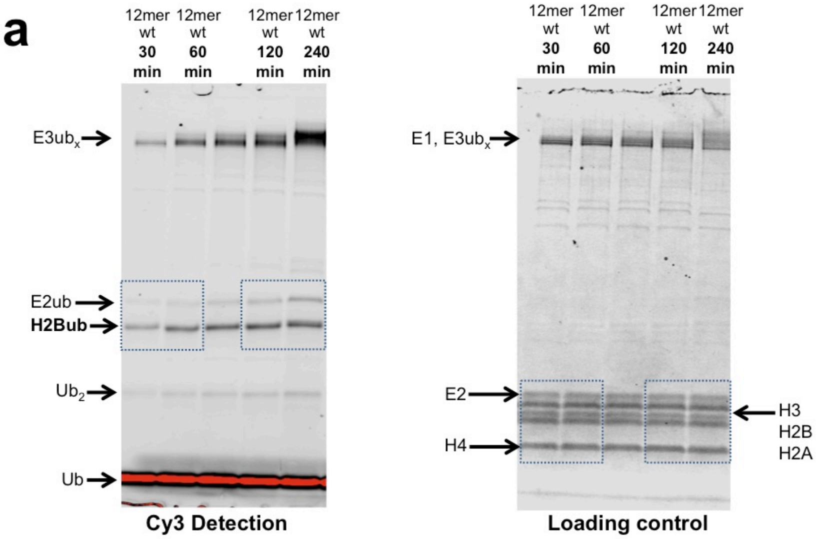

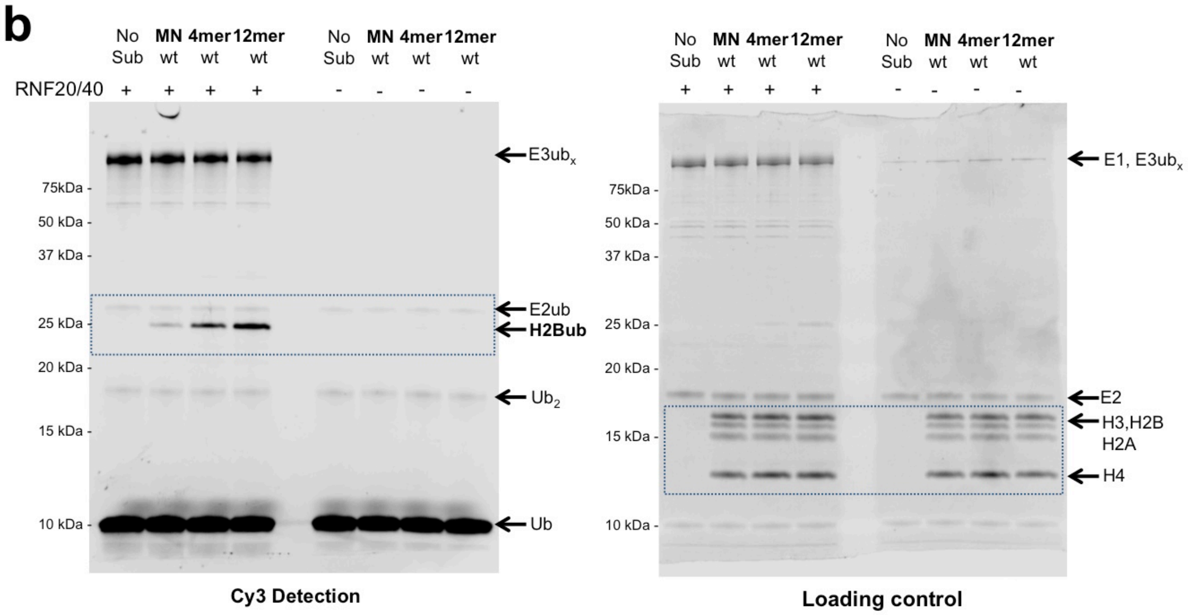

Supplementary Figure 17. Full images for data presented in Supplementary Fig. 4. Images were cropped for illustration purposes. (**a**) Full image of Cy3 readout and loading control (SYPRO Ruby protein stain) presented in Supplementary Fig. 4a. (**b**) Full image of Cy3 readout and loading control (SYPRO Ruby protein stain) presented in Supplementary Fig. 4b.

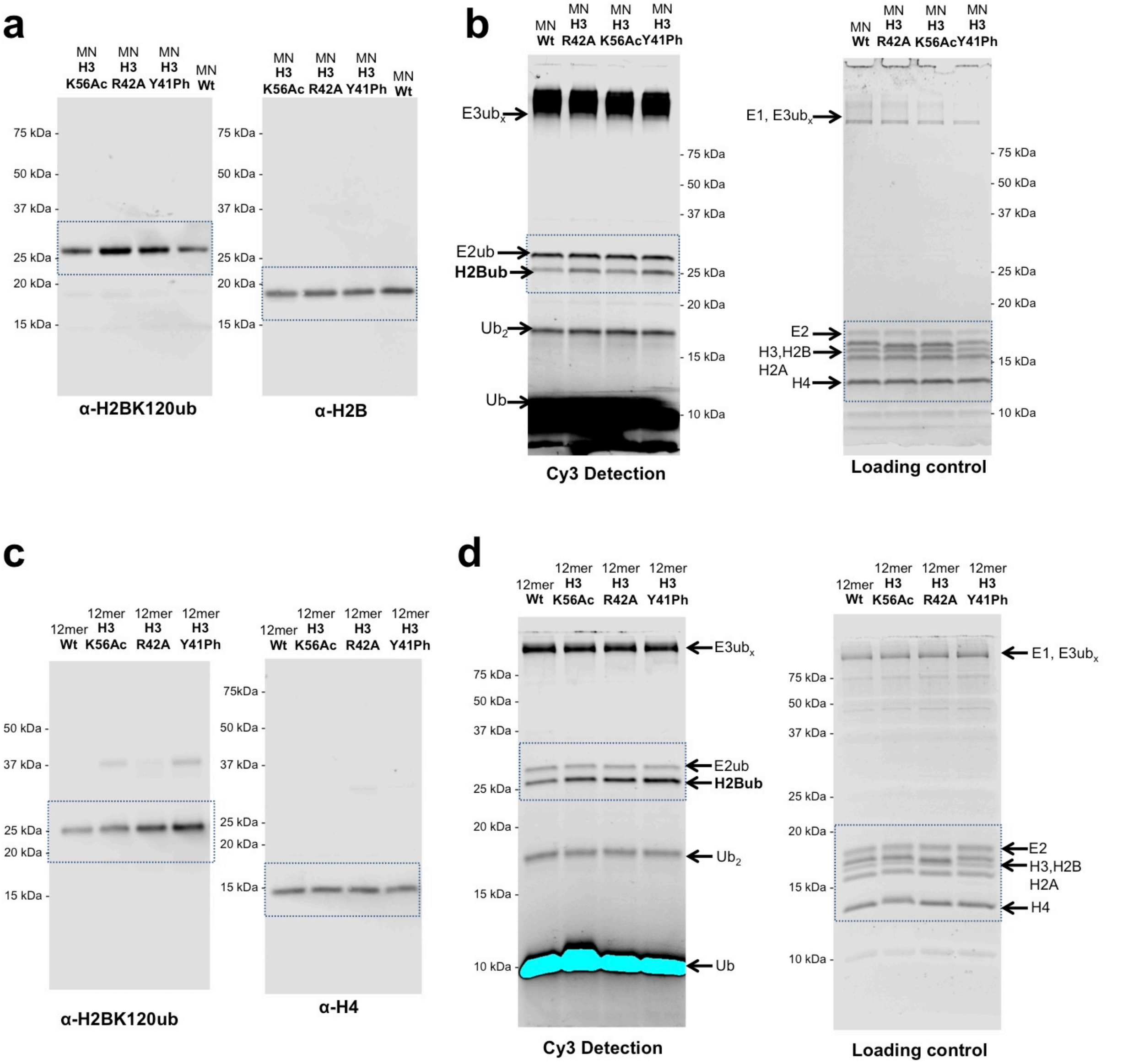

Supplementary Figure 18. Full images for data presented in Supplementary Fig. 5. Images were cropped for illustration purposes. (**a**) Full image for WB analysis presented in Supplementary Fig. 5b. (**b**) Full image of Cy3 readout and loading control (SYPRO Ruby protein stain) presented in Supplementary Fig. 5c. (**c**) Full image for WB analysis presented in Supplementary Fig. 5d. (**d**) Full image of Cy3 readout and loading control (SYPRO Ruby protein stain) presented in Supplementary Fig. 5e.

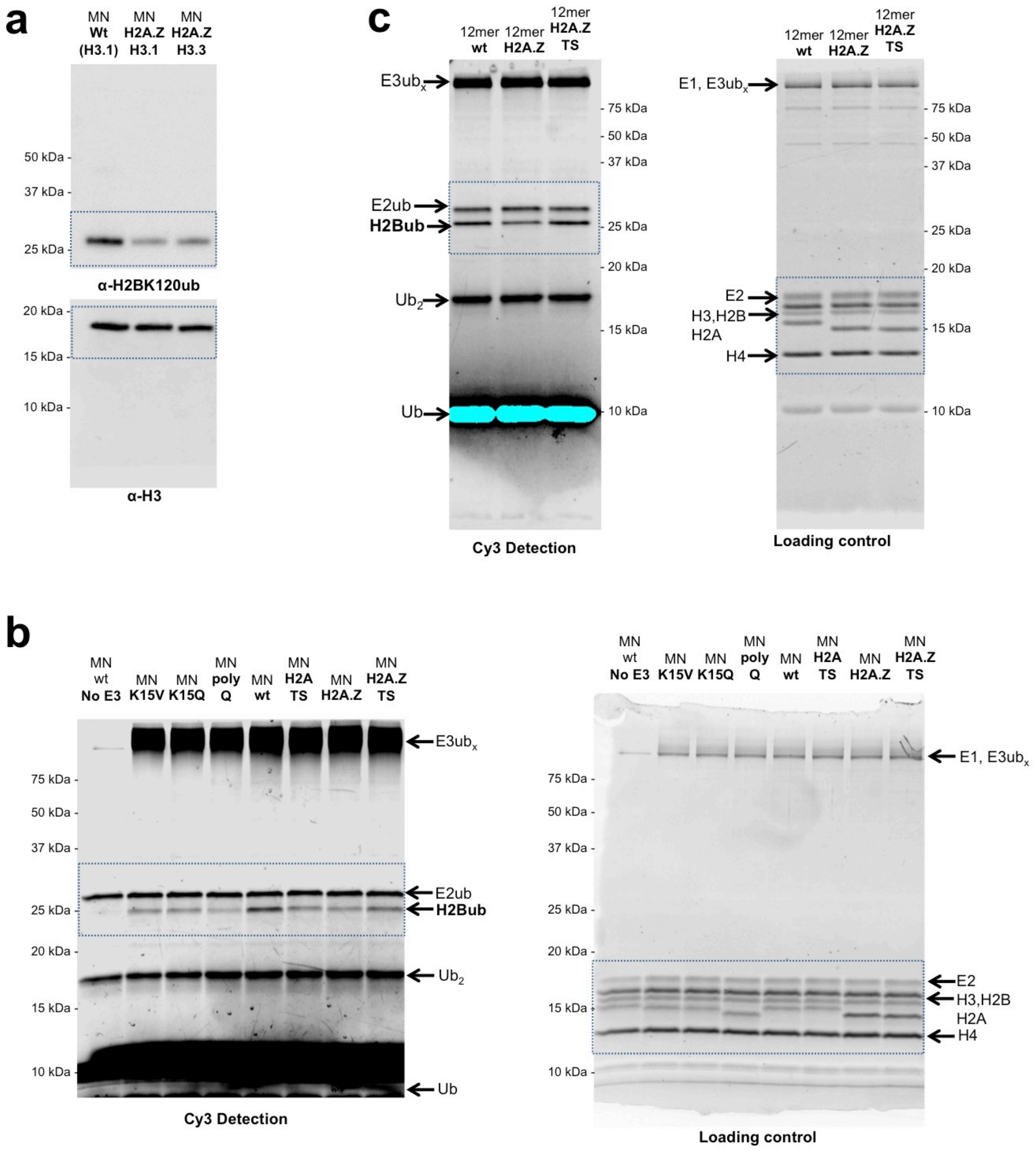

Supplementary Figure 19. Full images for data presented in Supplementary Fig. 7/9. Images were cropped for illustration purposes. (**a**) Full image for WB analysis presented in Supplementary Fig. 7b. WB was physically cut before antibody incubation. (**b**) Full image of Cy3 readout and loading control (SYPRO Ruby protein stain) presented in Supplementary Fig. 9a. (**c**) Full image of Cy3 readout and loading control (SYPRO Ruby protein stain) presented in Supplementary Fig. 9b.

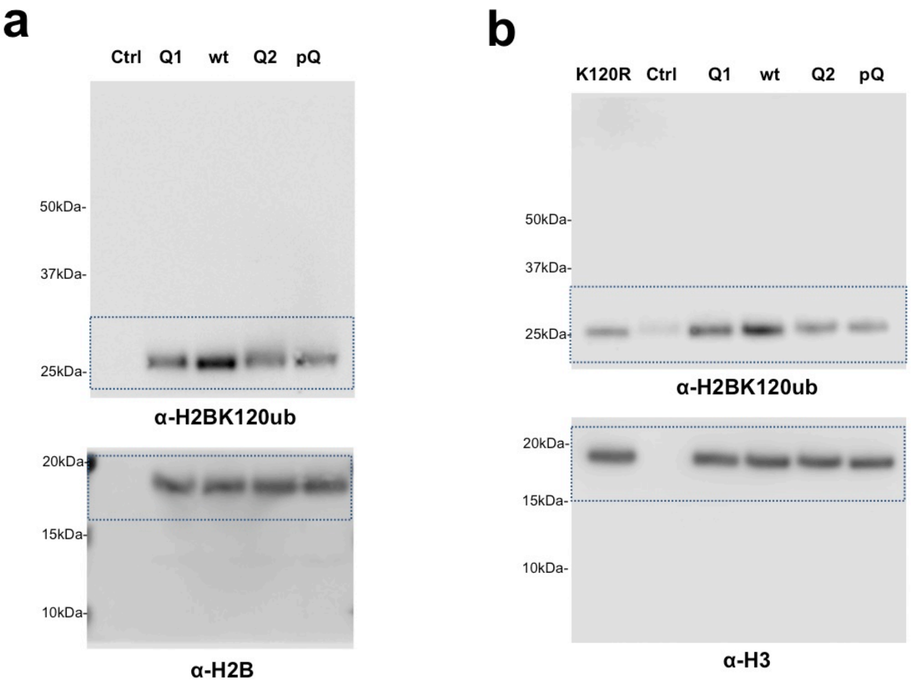

Supplementary Figure 20. Full images for data presented in Supplementary Fig. 10. Images were cropped for illustration purposes. (**a**) Full image for WB analysis presented in Supplementary Fig. 10c. WB was physically cut before antibody incubation. (**b**) Full image for WB analysis presented in Supplementary Fig. 10d. WB was physically cut before antibody incubation.

Supplementary Table 1. Library-based screen for *de novo* ubiquitylation activity. Substrates are ranked by *de novo* ubiquitylation activity from least active to most active. DNA reads were normalized to library input and the average signal intensities of the six wt-controls. Data are mean of $\log_2$(fold change) values ± s.e.m. (n = 3). Individual nucleosomes are color coded as in Fig. 2. Gray: Wt-controls; Red: Negative controls (pre-modified H2BK120); Light red: Free DNA; Dark blue: H2A N-terminal tail acetylation (single modification or combinations of K5Ac, K9Ac, K13Ac, K15Ac); Light blue: Nucleosomes containing histone variant H2A.Z; Orange: Entry/exit site modulators; Green: H2BS112GlcNAc. Rounded figures are displayed, the calculation of the mean and the s.e.m. was carried out with exact values.

							log2(fold change)	log2(fold change)	log2(fold change)	log2(fold change)	
	**Barcode**	**Histone H2A**	**Histone H2B**	**Histone H3**	**Histone H4**	**Modified**	**IP1**	**IP2**	**IP3**	**Average**	**SEM**
MN BC#116	CACTGT	H2AK5,9,13,15ac	H2B WT	H3 WT	H4 WT	H2A	-1.06	-0.60	-0.76	**-0.81**	0.13
MN BC#108	ACGCAG	H2A WT	H2BK120ac	H3 WT	H4 WT	H2B	-1.04	-0.50	-0.73	**-0.76**	0.16
MN BC#122	CTGCTA	H2AK5,9,13,15ac	H2BK5,11,12,16,20ac	H3K9,14,18,23,27ac	H4 WT	H2A, H2B, H3	-0.43	-0.57	-1.09	**-0.70**	0.20
MN BC#32	ACATGT	H2A WT	H2BK120ub	H3K9me3	H4 WT	H2B, H3	-1.17	-0.47	-0.41	**-0.68**	0.24
MN BC#135	ACGACT	H2A	H2BK120ub	H3 WT	H4R3me2a	H2B, H4	-1.08	-0.40	-0.56	**-0.68**	0.21
MN BC#31	CACAGT	H2A WT	H2BK120ub	H3K4me3	H4 WT	H2B, H3	-1.13	-0.35	-0.52	**-0.66**	0.24
MN BC#33	CGTCTG	H2A WT	H2BK120ub	H3K27me3	H4 WT	H2B, H3	-1.09	-0.45	-0.42	**-0.65**	0.22
MN BC#34	CTCTCG	H2A WT	H2BK120ub	H3K9,14,18,23,27ac	H4 WT	H2B, H3	-0.67	-0.19	-0.67	**-0.51**	0.16
MN BC#119	ACGTCG	H2A.Z	H2B WT	H3 WT	H4 WT	H2A	-0.72	-0.41	-0.40	**-0.51**	0.10
MN BC#120	CGAGAT	H2A.Z	H2B WT	H3.3	H4 WT	H2A, H3	-0.68	-0.37	-0.44	**-0.50**	0.09
MN BC#130	CTCTAT	H2AK15ac	H2B WT	H3 WT	H4 WT	H2A	-0.73	-0.42	-0.34	**-0.50**	0.12
MN BC#547	ACGTCT	x	x	x	x	free DNA no PST	-0.71	-0.07	-0.45	**-0.41**	0.19
MN BC#40	CTCAGT	H2A WT	H2B WT	H3K9,14,18,23,27ac	H4K5,8,12,16,20ac	H3, H4	0.13	-0.42	-0.87	**-0.39**	0.29
MN BC#121	ATCACG	H2AK5,9,13,15ac	H2BK5,11,12,16,20ac	H3K9,14,18,23,27ac	H4K5,8,12,16,20ac	H2A, H2B, H3, H4	-0.13	-0.36	-0.65	**-0.38**	0.15
MN BC#129	GCGCTG	H2AK13ac	H2B WT	H3 WT	H4 WT	H2A	-0.35	-0.28	-0.23	**-0.29**	0.03
MN BC#21	CATGAT	H2A WT	H2B WT	H3K9me3	H4K5,8,12,16,20ac	H3, H4	-0.16	-0.32	-0.28	**-0.25**	0.05
MN BC#136	AGCTCG	H2AK119ub	H2B WT	H3 WT	H4R3me2a	H2A, H4	-0.33	-0.15	-0.27	**-0.25**	0.05
MN BC#18	AGCGTA	H2A WT	H2B WT	H3K9me3	H4K12ac	H3, H4	-0.15	-0.26	-0.24	**-0.22**	0.03
MN BC#30	AGAGCA	H2A WT	H2BK120ub	H3 WT	H4 WT	H2B	-0.29	0.00	-0.35	**-0.21**	0.11
MN BC#3	CAGTAT	H2A WT	H2B WT	H3 WT	H4K8ac	H4	-0.22	-0.20	-0.20	**-0.21**	0.01
MN BC#14	GTATCT	H2A WT	H2B WT	H3K4me3	H4K5,8,12,16,20ac	H3, H4	-0.08	-0.28	-0.27	**-0.21**	0.07
MN BC#109	AGCATA	H2A WT	H2BK125ac	H3 WT	H4 WT	H2B	-0.24	-0.08	-0.21	**-0.17**	0.05
MN BC#96	GCAGCA	H2A WT	H2B WT	H3K79ac	H4 WT	H3	-0.22	-0.12	-0.16	**-0.17**	0.03
MN BC#5	ACGTGA	H2A WT	H2B WT	H3 WT	H4K16ac	H4	-0.15	-0.17	-0.16	**-0.16**	0.01
MN BC#6	CTCACG	H2A WT	H2B WT	H3 WT	H4K20ac	H4	-0.17	-0.20	-0.09	**-0.16**	0.03
MN BC#28	AGCACG	H2A WT	H2B WT	H3K27me3	H4K5,8,12,16,20ac	H3, H4	-0.05	-0.16	-0.25	**-0.15**	0.06
MN BC#16	ATGTGT	H2A WT	H2B WT	H3K9me3	H4K5ac	H3, H4	0.01	-0.24	-0.22	**-0.15**	0.08
MN BC#111	GTATAT	H2AK5ac	H2B WT	H3 WT	H4 WT	H2A	-0.13	-0.12	-0.18	**-0.14**	0.02
MN BC#20	CTGCGA	H2A WT	H2B WT	H3K9me3	H4K20ac	H3, H4	-0.09	-0.12	-0.17	**-0.13**	0.02
MN BC#17	GCTCGA	H2A WT	H2B WT	H3K9me3	H4K8ac	H3, H4	-0.09	-0.13	-0.12	**-0.11**	0.01
MN BC#36	CTATAG	H2AK119ub	H2B WT	H3 WT	H4 WT	H2A	-0.22	-0.07	-0.05	**-0.11**	0.06
MN BC#10	CATACA	H2A WT	H2B WT	H3K4me3	H4K8ac	H3, H4	-0.14	-0.04	-0.14	**-0.11**	0.03
MN BC#2	ACGACG	H2A WT	H2B WT	H3 WT	H4K5ac	H4	-0.02	-0.12	-0.18	**-0.11**	0.05
MN BC#106	CGATCG	H2A WT	H2BK108ac	H3 WT	H4 WT	H2B	-0.10	-0.05	-0.15	**-0.10**	0.03
MN BC#112	GCTGAG	H2AK9ac	H2B WT	H3 WT	H4 WT	H2A	-0.12	-0.05	-0.14	**-0.10**	0.03
MN BC#12	CAGAGA	H2A WT	H2B WT	H3K4me3	H4K16ac	H3, H4	-0.02	-0.05	-0.24	**-0.10**	0.07
MN BC#64	CACACA	H2A WT	H2B WT	H3 WT	H4R17A,R19A	H4	-0.10	-0.04	-0.13	**-0.09**	0.03
MN BC#19	ATACAG	H2A WT	H2B WT	H3K9me3	H4K16ac	H3, H4	-0.02	-0.12	-0.14	**-0.09**	0.04
MN BC#98	AGATCA	H2A WT	H2B WT	H3K122ac	H4 WT	H3	-0.15	-0.06	-0.06	**-0.09**	0.03
MN BC#75	CGCGCG	H2A WT	H2B WT	H3 WT	H4K91ac	H4	-0.09	-0.02	-0.17	**-0.09**	0.04
MN BC#127	ACTGCT	H2A WT	H2B WT	H3T118H	H4R17A,R19A	H3, H4	-0.04	-0.09	-0.12	**-0.08**	0.02
MN BC#15	GAGCAG	H2A WT	H2B WT	H3K9me3	H4 WT	H3	-0.09	-0.09	-0.03	**-0.07**	0.02
MN BC#7	GTGACA	H2A WT	H2B WT	H3 WT	H4K5,8,12,16,20ac	H4	0.05	-0.11	-0.15	**-0.07**	0.06
MN BC#43	GTAGCT	H2A WT	H2B WT	H3K18ac	H4 WT	H3	0.02	-0.01	-0.18	**-0.06**	0.06
MN BC#23	GCTCAG	H2A WT	H2B WT	H3K27me3	H4K5ac	H3, H4	0.00	-0.10	-0.05	**-0.05**	0.03
MN BC#82	GATGCT	H2A WT	H2B WT	H3.3K27M	H4 WT	H3	0.07	0.03	-0.25	**-0.05**	0.10
MN BC#9	AGACTA	H2A WT	H2B WT	H3K4me3	H4K5ac	H3, H4	-0.03	-0.09	-0.01	**-0.05**	0.02
MN BC#4	ACAGTA	H2A WT	H2B WT	H3 WT	H4K12ac	H4	-0.11	-0.05	0.02	**-0.04**	0.04
MN BC#11	GCACAG	H2A WT	H2B WT	H3K4me3	H4K12ac	H3, H4	-0.04	-0.11	0.02	**-0.04**	0.04
MN BC#1	CGAGAG	x	x	x	x	free DNA +PST	-0.04	0.34	-0.42	**-0.04**	0.22
MN BC#8	GCGACA	H2A WT	H2B WT	H3K4me3	H4 WT	H3	-0.01	-0.05	-0.06	**-0.04**	0.02
MN BC#26	GTATGA	H2A WT	H2B WT	H3K27me3	H4K16ac	H3, H4	0.04	-0.02	-0.12	**-0.04**	0.05
MN BC#342	CGCGCA	H2A WT	H2B WT	H3 WT	H4 WT	ALL WT	-0.03	0.01	-0.08	**-0.03**	0.02
MN BC#24	CATCTA	H2A WT	H2B WT	H3K27me3	H4K8ac	H3, H4	0.05	0.00	-0.15	**-0.03**	0.06
MN BC#63	CGACGT	H2A WT	H2B WT	H3 WT	H4 WT	ALL WT	-0.03	-0.02	-0.04	**-0.03**	0.01
MN BC#344	GCAGTG	H2A WT	H2B WT	H3 WT	H4 WT	ALL WT	-0.04	0.02	-0.04	**-0.02**	0.02
MN BC#48	CACTGA	H2A WT	H2B WT	H3K18acK23ac	H4 WT	H3	0.07	0.01	-0.14	**-0.02**	0.06
MN BC#128	ACACTA	H2A WT	H2B WT	H3 WT	H4 WT	CpG	-0.01	-0.03	-0.02	**-0.02**	0.01
MN BC#44	CAGTCT	H2A WT	H2B WT	H3K23ac	H4 WT	H3	0.05	0.07	-0.16	**-0.02**	0.07
MN BC#139	ACGTAG	H2A WT	H2B WT	H3K18cr	H4 WT	H3	0.13	0.00	-0.18	**-0.02**	0.09
MN BC#105	ATCGCA	H2A WT	H2BK5,11,12,16,20ac	H3 WT	H4 WT	H2B	0.02	0.03	-0.09	**-0.01**	0.04
MN BC#89	GTCTCA	H2A WT	H2B WT	H3S10ph	H4 WT	H3	-0.02	0.08	-0.09	**-0.01**	0.05
MN BC#137	ATGTAT	H2A WT	H2B WT	H3K9cr	H4 WT	H3	0.07	0.07	-0.16	**0.00**	0.08
MN BC#138	GACGCG	H2A WT	H2B WT	H3K14cr	H4 WT	H3	0.01	-0.02	0.00	**0.00**	0.01
MN BC#13	ATATCA	H2A WT	H2B WT	H3K4me3	H4K20ac	H3, H4	0.02	-0.04	0.04	**0.01**	0.02
MN BC#351	ATACTG	H2A WT	H2B WT	H3 WT	H4 WT	ALL WT	0.01	0.02	-0.01	**0.01**	0.01
MN BC#107	GTACGA	H2A WT	H2BK116ac	H3 WT	H4 WT	H2B	0.08	0.11	-0.17	**0.01**	0.09
MN BC#132	GTACTA	H2A WT	H2B WT	H3K4me1	H4 WT	H3	0.08	0.05	-0.10	**0.01**	0.05
MN BC#45	GTGAGT	H2A WT	H2B WT	H3K27ac	H4 WT	H3	0.14	0.01	-0.09	**0.02**	0.07
MN BC#42	CTGTCG	H2A WT	H2B WT	H3K14ac	H4 WT	H3	0.05	0.08	-0.06	**0.02**	0.04
MN BC#125	CGTAGA	H2A WT	H2B WT	H3T118H	H4R45A	H3, H4	0.10	0.07	-0.11	**0.02**	0.07
MN BC#41	CGACGA	H2A WT	H2B WT	H3K9ac	H4 WT	H3	0.08	0.07	-0.08	**0.02**	0.05
MN BC#104	AGTGAT	H2A WT	H2BK20ac	H3 WT	H4 WT	H2B	0.06	0.09	-0.08	**0.02**	0.05
MN BC#352	ATATAT	H2A WT	H2B WT	H3 WT	H4 WT	ALL WT	0.03	-0.04	0.09	**0.03**	0.04
MN BC#67	GACTAG	H2A WT	H2B WT	H3 WT	H4R3me2a	H4	0.12	0.09	-0.09	**0.04**	0.07
MN BC#133	ATGCGA	H2A WT	H2B WT	H3K4me2	H4 WT	H3	0.07	0.01	0.05	**0.04**	0.02
MN BC#27	ATCGTG	H2A WT	H2B WT	H3K27me3	H4K20ac	H3, H4	0.05	0.06	0.03	**0.05**	0.01
MN BC#350	ACATCA	H2A WT	H2B WT	H3 WT	H4 WT	ALL WT	0.06	0.02	0.07	**0.05**	0.02
MN BC#100	ATGTCA	H2A WT	H2BK11ac	H3 WT	H4 WT	H2B	0.08	0.02	0.05	**0.05**	0.02

Least active substrates shown in Figure 2B

MN BC#99	CGAGTA	H2A WT	H2BK5ac	H3 WT	H4 WT	H2B	0.13	0.15	-0.12	**0.05**	0.09
MN BC#84	GTGCTA	H2A WT	H2B WT	H3K36me3	H4 WT	H3	0.09	0.07	0.01	**0.05**	0.02
MN BC#22	AGCTGT	H2A WT	H2B WT	H3K27me3	H4 WT	H3	0.07	0.06	0.03	**0.06**	0.01
MN BC#74	GCGTGA	H2A WT	H2B WT	H3 WT	H4K79ac	H4	0.13	0.08	-0.02	**0.06**	0.04
MN BC#25	CTCTCA	H2A WT	H2B WT	H3K27me3	H4K12ac	H3, H4	0.11	0.09	0.01	**0.07**	0.03
MN BC#97	AGATAG	H2A WT	H2B WT	H3K115ac	H4 WT	H3	0.19	0.08	-0.06	**0.07**	0.07
MN BC#87	GCACGA	H2A WT	H2B WT	H3R2me	H4 WT	H3	0.11	0.11	0.02	**0.08**	0.03
MN BC#140	CTATGT	H2A WT	H2B WT	H3K27cr	H4 WT	H3	0.28	0.01	-0.05	**0.08**	0.10
MN BC#102	CGTCGA	H2A WT	H2BK15ac	H3 WT	H4 WT	H2B	0.13	0.13	-0.02	**0.08**	0.05
MN BC#113	GAGTCA	H2AK118ac	H2B WT	H3 WT	H4 WT	H2A	0.21	0.06	-0.02	**0.08**	0.07
MN BC#29	CGCGAT	H2A WT	H2B WT	H3K9,14,18,23,27ac	H4 WT	H3	0.35	0.11	-0.19	**0.09**	0.16
MN BC#73	CGCACG	H2A WT	H2B WT	H3 WT	H4K77ac	H4	0.15	0.14	0.00	**0.10**	0.05
MN BC#91	CACACG	H2A WT	H2B WT	H3K4ac	H4 WT	H3	0.23	0.15	-0.07	**0.10**	0.09
MN BC#101	CTCGTG	H2A WT	H2BK12ac	H3 WT	H4 WT	H2B	0.14	0.14	0.02	**0.10**	0.04
MN BC#78	AGCTAG	H2A WT	H2B WT	H3.3	H4 WT	H3	0.15	0.11	0.07	**0.11**	0.02
MN BC#81	CTCGAT	H2A WT	H2B WT	H3.3G34W	H4 WT	H3	0.13	0.18	0.05	**0.12**	0.04
MN BC#80	ATCTAT	H2A WT	H2B WT	H3.3G34R	H4 WT	H3	0.12	0.13	0.11	**0.12**	0.01
MN BC#79	ATGACT	H2A WT	H2B WT	H3.3G34V	H4 WT	H3	0.14	0.13	0.12	**0.13**	0.00
MN BC#118	ACAGAG	H2A.X	H2B WT	H3 WT	H4 WT	H2A	0.27	0.15	-0.02	**0.14**	0.08
MN BC#86	GTGCAT	H2A WT	H2B WT	H3K4me3K27me3	H4 WT	H3	0.35	0.16	0.04	**0.18**	0.09
MN BC#77	ACTAGA	H2A WT	H2B WT	H3T118H	H4 WT	H3	0.23	0.29	0.04	**0.19**	0.07
MN BC#65	ATATAG	H2A WT	H2B WT	H3 WT	H4R45A	H4	0.27	0.16	0.18	**0.20**	0.03
MN BC#88	CGTATA	H2A WT	H2B WT	H3R2me2a	H4 WT	H3	0.22	0.20	0.21	**0.21**	0.01
MN BC#95	AGCATG	H2A WT	H2B WT	H3K64ac	H4 WT	H3	0.28	0.23	0.16	**0.22**	0.03
MN BC#92	CACTCG	H2A WT	H2B WT	H3K36ac	H4 WT	H3	0.34	0.28	0.10	**0.24**	0.07
MN BC#103	GCAGTA	H2A WT	H2BK16ac	H3 WT	H4 WT	H2B	0.35	0.25	0.15	**0.25**	0.06
MN BC#90	GAGTCT	H2A WT	H2B WT	H3R42me2a	H4 WT	H3	0.33	0.22	0.21	**0.25**	0.04
MN BC#93	GCGAGA	H2A WT	H2B WT	H3K37ac	H4 WT	H3	0.44	0.36	0.13	**0.31**	0.09
MN BC#68	ACATGA	H2A WT	H2B WT	H3 WT	H4R3me2s	H4	0.42	0.35	0.22	**0.33**	0.06
MN BC#134	AGATGT	H2A	H2BS112GlcNAc	H3 WT	H4	H2B	0.51	0.32	0.38	**0.40**	0.06
MN BC#83	AGTAGA	H2A WT	H2B WT	H3.3K36M	H4 WT	H3	0.51	0.42	0.31	**0.41**	0.06
MN BC#66	AGCTCT	H2A WT	H2B WT	H3 WT	H4R3me	H4	0.47	0.49	0.34	**0.44**	0.05
MN BC#94	GTCTAG	H2A WT	H2B WT	H3K56ac	H4 WT	H3	0.62	0.45	0.27	**0.45**	0.10
MN BC#126	ATCATG	H2A WT	H2B WT	H3R42A	H4R17A,R19A	H3, H4	0.64	0.47	0.48	**0.53**	0.05
MN BC#76	CAGCTG	H2A WT	H2B WT	H3R42A	H4 WT	H3	0.80	0.56	0.53	**0.63**	0.08
MN BC#124	GTCTAT	H2A WT	H2B WT	H3R42A	H4R45A	H3, H4	0.88	0.58	0.53	**0.66**	0.11
MN BC#85	ATATGA	H2A WT	H2B WT	H3Y41ph	H4 WT	H3	1.41	1.09	1.05	**1.18**	0.12

Most active substrates shown in Figure 2B

Supplementary Table 2. List of antibodies used in this study.

Epitope	Vendor (product #)	Antibody dilution	Application
Anti-H3	Abcam (Ab1791)	1:10000 in TBS-T	Loading control
Anti-H2B	Abcam (Ab1790)	1:10000 in TBS-T	Loading control
Anti-H4	Abcam (Ab31830)	1:2000 in TBS-T	Loading control
Anti-H2BK120ub	Medimabs (MM0029)	1:1000 in TBS-T	*In vitro* ubiquitylation levels
Anti-H2BK120ub	CST (Ubiquityl-Histone H2B XP® Rabbit mAb #5546)	1:1000 in TBS-T containing 5% BSA	*In vitro* ubiquitylation levels and cellular ubiquitylation levels
Anti-HA	Covance (anti-HA.11 Clone 16B12, MMS-101R)	See method section	*In vitro* ChIP of the MN-Library
Anti-FLAG	Sigma Aldrich (M2 clone, F3165)	See method section	ChIP of cellular MNs
Anti-RNF20	Abcam (Ab32629)	1:1000 in TBS-T	Autoubiquitylation of RNF20
Anti-Rabbit	Bio-Rad HRP conjugate (170-6515)	1:10000 in TBS-T	Visualization of prim. antibody
Anti-Mouse	Bio-Rad HRP conjugate (170-6516)	1:10000 in TBS-T	Visualization of prim. antibody
Anti-Rabbit	LI-COR (IRDye@800CW 926-32211)	1:15000 in TBS-T	Visualization of prim. antibody
Anti-Rabbit	LI-COR (IRDye@680LT 877-11081)	1:15000 in TBS-T	Visualization of prim. antibody
Anti-Mouse	LI-COR (IRDye@800CW 827-08364)	1:15000 in TBS-T	Visualization of prim. antibody

Supplementary Table 3. Summary of ChIP-seq and RNA-seq datasets used in this study.

Dataset	Cell type	Data source	Laboratory responsible for data generation	Accession number
H2A.Z ChIP-seq	HeLa-S3	ENCODE consortium	Bradley Bernstein, Broad Institute	ENCFF532VFI
H3K79me2 ChIP-seq	HeLa-S3	ENCODE consortium	Bradley Bernstein, Broad Institute	ENCFF432DSJ
H3K4me3 ChIP-seq	HeLa-S3	ENCODE consortium	Bradley Bernstein, Broad Institute	ENCFF699TXY
Strand-specific, rRNA-depleted, poly(A)+ RNA-seq	HeLa-S3	ENCODE consortium	Thomas Gingeras, CSHL	ENCFF000FNX, ENCFF000FNY
Strand-specific, rRNA-depleted, poly(A)+ RNA-seq	HeLa-S3	ENCODE consortium	Thomas Gingeras, CSHL	ENCFF084ARU
MNase-seq	HeLa-S3	European Nucleotide Archive	Giuseppe Macino, Sapienza University of Rome	ERS345758
H2BK120ub ChIP-seq	HeLa	NCBI Gene Expression Omnibus	Didier Devys, Institut de Génétique et de Biologie Moléculaire et Cellulaire	GSM1277116
